# Repeat Faecal Immunochemical Testing for Colorectal Cancer Detection in Symptomatic and Screening Patients: A Systematic Review and Meta-Analysis

**DOI:** 10.3390/cancers16183199

**Published:** 2024-09-19

**Authors:** Adam D. Gerrard, Roberta Garau, Wei Xu, Yasuko Maeda, Malcolm G. Dunlop, Evropi Theodoratou, Farhat V. N. Din

**Affiliations:** 1 Cancer Research UK Scotland Centre, Institute of Genetics and Cancer, The University of Edinburgh, Edinburgh EH4 2XR, UK; adam.gerrard@ed.ac.uk (A.D.G.); e.theodoratou@ed.ac.uk (E.T.); 2 Department of Colorectal Surgery, Western General Hospital, Edinburgh EH4 2XU, UK; 3 Centre for Global Health, Usher Institute, The University of Edinburgh, Edinburgh EH4 2XR, UK; 4 School of Medicine, Dentistry and Nursing, University of Glasgow, Glasgow G12 8QQ, UK; 5 Department of Surgery, Queen Elizabeth University Hospital, Glasgow G51 4TF, UK; 6 UK Colon Cancer Genetics Group, Medical Research Council Human Genetics Unit, Medical Research Council Institute of Genetics & Cancer, Western General Hospital, The University of Edinburgh, Edinburgh EH4 2XU, UK

**Keywords:** faecal immunochemical testing, FIT, colorectal cancer, CRC, colorectal diagnosis, bowel screening

## Abstract

**Simple Summary:**

Faecal immunochemical testing is used to help aid the detection of bowel cancers and other serious bowel problems. It is a stool test that looks for non-visible blood. It has been shown that doing more than one test may improve the rate of detection of serious bowel problems; however, the evidence for this is not well described and has not been reviewed. We aimed to review the available data for using more than one test in different risk groups, exploring the effect on positivity and workload for additional tests along with assessing how well multiple tests work at detecting bowel cancer and other problems. The results of this are important as they help in the development of services to detect colorectal cancer.

**Abstract:**

Background: Faecal immunochemical testing (FIT) is widely used in bowel screening programmes and assessing symptomatic patients for suspected colorectal cancer (CRC). The evidence for single test performance of FIT in both settings is considerable; however, the use of a repeat test to increase sensitivity remains uncertain. We aimed to review what increase in test positivity would be generated by additional FITs, whether a repeated FIT detects previously missed CRC and advanced colorectal neoplasia (ACRN), and to estimate the sensitivity of double-FIT strategies to diagnose CRC and ACRN. Methods: A systematic search of MEDLINE, EMBASE, and the Cochrane Central Register of Controlled Trials (CENTRAL) was performed using key search terms. Studies reporting the use of more than one FIT in the same screening round or planned assessment of a single symptomatic patient episode were included. Studies were categorised by the reported study population into asymptomatic, mixed (cohorts of combined asymptomatic, symptomatic, or high-risk surveillance), or symptomatic cohorts. Results: A total of 68 studies were included for analysis (39 asymptomatic, 21 mixed, 7 symptomatic, and 1 study with discrete asymptomatic and symptomatic data). At a threshold of 10 µg Hb/g, the two-test positivity ranged between 8.1 and 34.5%, with an increase from the second test of 3–9.2 percentage points. Four out of five studies comparing one versus two tests for diagnosing CRC at 10 µg Hb/g identified additional cases with the second test, with a minimum of 50% reduction in missed CRC. At a threshold of 20 µg Hb/g, the second test increased the positivity by 1.3–6.7 percentage points, with a two-test positivity of between 5.1 and 25.0%. Using a threshold of 20 µg Hb/g, five out of seven studies had a 25% reduction in missed CRC. A meta-analysis estimated the double-FIT sensitivity at 10 µg Hb/g for CRC in mixed-risk and symptomatic cohorts to be 94% and 98%, respectively. Conclusions: Repeated use of FIT helps to diagnose more cases of CRC with a moderate increase in positivity. A double-FIT strategy at 10 µg Hb/g in mixed and symptomatic cohorts has a very high sensitivity for CRC.

## 1. Introduction

Faecal immunochemical testing (FIT) has become the established method of non-invasive bowel cancer screening worldwide [1], and more recently, adopted as front-line investigation for patients with symptoms suggestive of colorectal cancer (CRC) [2]. FIT may be qualitative, with a fixed value for positivity, or quantitative, where thresholds can be set depending on available resources and predictive diagnostic yield.

The use of a single FIT in screening and symptomatic cohorts has been an area of emergent development over recent years. Contemporary meta-analyses estimate the single-FIT sensitivity for CRC to be 67% in screening programmes with a positivity threshold of 40 µg haemoglobin per gram (Hb/g) or less [3], and between 88 and 90% at 10 µg Hb/g in symptomatic cohorts [3,4,5]. Therefore, there is scope to improve test sensitivity, which may be achieved by using more than one test.

Utilising multiple FITs during the same screening round or assessment of symptoms has not been as widely studied. A previous review containing seven studies of both qualitative and quantitative FIT found any benefit to repeat testing to be inconclusive [6]. This study did not separate the populations being studied by risk or aim to analyse positivity and sensitivity at reported thresholds. After the publication of the review, there has been an increasing interest in using more than one FIT to reduce cases of missed significant bowel pathology [7,8].

We therefore aimed to perform a systematic literature review for the utilisation of more than one FIT in asymptomatic, mixed, and symptomatic cohorts. Assessment of the workload generated by positivity rates and the benefits of reducing missed CRC and advanced colorectal neoplasia (ACRN; CRC and advanced adenomas) were estimated. A meta-analysis was performed to estimate the sensitivity of double-FIT in the defined cohorts.

## 2. Methods

A systematic review of the literature and a meta-analysis were conducted, investigating the use of multiple FITs for the detection of CRC and ACRN in asymptomatic, mixed, and symptomatic populations. The protocol was registered with the International Prospective Review of Systematic Reviews (PROSPERO CRD42020207219). The review conformed to the Preferred Reporting Items for Systematic Reviews and Meta-Analyses (PRISMA) statement standards [9].

### 2.1. Review Questions

1. Is the test positivity rate higher with multiple FITs compared with single FITs within the study populations?
2. Is the rate of missed CRC and ACRN decreased with the addition of second and/or third FIT tests compared with single FITs?
3. In asymptomatic, mixed, and symptomatic populations, what is the sensitivity for CRC and ACRN detection when a double-FIT strategy is used at key thresholds?

### 2.2. Data Search Strategy

Studies were identified through searches of MEDLINE, EMBASE, and Cochrane Central Register of Controlled Trials databases from January 2000 up to December 2022. Studies published prior to 2000 were excluded due to advances in FIT technology. The search was updated before the analysis in March 2023. The search strategy is outlined in Supplementary File S1. Reference lists of relevant articles and reviews were searched for further potentially eligible studies.

### 2.3. Study Selection

Inclusion criteria for the studies were as follows: (1) use of faecal immunochemical tests, (2) published after 2000, (3) planned use of multiple tests on different bowel motions within a month of each test (this was selected to ensure tests were being performed for the same indication), (4) outcome data for test performance at diagnosis of CRC or ACRN, (5) data of FIT used with thresholds considered positive, and (6) for review questions 2 and 3, studies were only included if they used quantitative FITs and were able to report complete true positive, false positive, true negative, or false negative results, or if they could be calculated from published data, with complete investigation or registry follow-up. Exclusion criteria were (1) non-full text articles, (2) studies not published in English, (3) studies published before 2000, (4) studies where multiple FITs were performed on the same stool, (5) cost modelling with no novel test performance data, and (6) the use of a single FIT over multiple rounds of screening/surveillance. Where multiple studies contained the same, or partial, data, the most recent and/or complete data were used. Two authors independently assessed the titles and abstracts of articles identified through the literature searches. Full texts of relevant articles were retrieved, and those meeting the study criteria were selected again by the two authors. Where a discrepancy occurred, resolution was sought first by discussion between the authors, and in the case of disagreement, a third author’s opinion was sought.

### 2.4. Data Extraction and Assessment

An electronic data extraction spreadsheet was made. This was pilot-tested using ten randomly selected articles. Where possible, FIT thresholds used in studies were standardised to µg Hb/g using available published data and manufacturer information [10,11]. Data extraction was performed by one author and verified by a second. Study populations were categorised into asymptomatic, mixed, or symptomatic based on their individual inclusion criteria. Where articles were not purely average risk asymptomatic screening, or not all patients were symptomatic of suspected colonic pathology, the study was classified as a mixed-population study. Included studies were appraised using the QUADAS-2 tool (Supplementary File S2) [12]. Positivity data were extracted at key thresholds for one (first) test, positivity when using two tests, and where applicable, three tests. The decrease in missed CRC and ACRN with a second or third test was calculated in studies that used quantitative FIT, investigated or had registry outcomes for all patients, and reported first-test and multiple-test performance. The first reported threshold was 10 µg Hb/g as this was the threshold in symptomatic patients suggested by NICE and ACPGBI guidance [2,13]. Second thresholds were selected where multiple studies reported data at the same thresholds and analysis could be conducted. Overall diagnostic performance was assessed in studies with full investigations or registry outcomes where a two-test strategy had been used. To ensure sufficient power to the results, where four or more of these studies within the same population group reported results at key thresholds, a meta-analysis was performed.

We pooled together sensitivity and specificity using the random-effects model (DerSimonian–Laird) [14]. Heterogeneity among studies was tested using Cochran’s Q statistic, the I^2^ index, and the tau-squared test [15]. Funnel plots and the Egger test were used to detect evidence of publication bias (Supplementary File S3) [16]. A two-sided *p*-value of less than 0.05 was considered statistically significant. The meta-analyses were conducted using the ‘meta’ R package. All analyses were performed using R, version 4.0.3 (R Foundation for Statistical Computing, Vienna, Austria).

### 2.5. Outcome Measures

Positivity rates were collected from included studies at given thresholds. One test (first test), that the patient performs, two test (at least one positive), and three test (at least one positive) rates were utilised, and the percentage point increase between the first and subsequent tests was calculated. For studies meeting the criteria for diagnostic performance assessment, those reporting outcomes from one test and multiple tests were analysed. ‘Missed’ cases (false negatives) were compared between one and two tests to investigate the relative percentage reduction in missed pathology by additional tests. Where more than four studies of the same population and threshold report sensitivity data for CRC and/or ACRN, a meta-analysis was performed to estimate the sensitivity of a double-FIT strategy.

## 3. Results

**Figure 1:**
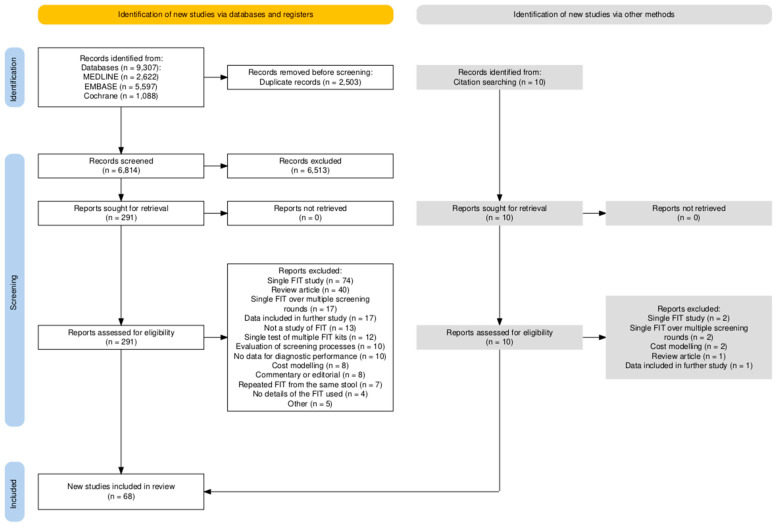
PRISMA study flow diagram.

The literature search of the databases and references identified 6814 unique articles. After screening for titles and abstracts, 301 full texts were reviewed, and finally, 68 studies were included (Figure 1). Studies that utilised multiple FITs but did not meet inclusion criteria can be found in Supplementary File S4. The population under investigation was asymptomatic screening in 39 studies [17,18,19,20,21,22,23,24,25,26,27,28,29,30,31,32,33,34,35,36,37,38,39,40,41,42,43,44,45,46,47,48,49,50,51,52,53,54,55], a mixed population of screening, surveillance, and symptoms in 21 studies [56,57,58,59,60,61,62,63,64,65,66,67,68,69,70,71,72,73,74,75,76], and 7 studies that reported wholly symptomatic cohort results [7,8,77,78,79,80,81]. One study contained separate data on both asymptomatic and symptomatic cohorts that could be analysed in their respective populations (Table 1) [82]. Details of the included studies can be found in Supplementary File S5. Of the included studies, 23 employed a qualitative FIT [17,18,19,20,21,33,34,35,36,45,47,52,53,55,61,62,65,68,72,73,75,78,82] and 45 used a quantitative method [7,8,22,23,24,26,27,28,29,30,31,32,37,38,39,40,41,42,43,44,46,48,49,50,51,56,57,58,59,60,63,64,66,67,69,70,71,74,76,77,79,80,81,83,84].

### 3.1. Positivity Rates

**Figure 2:**
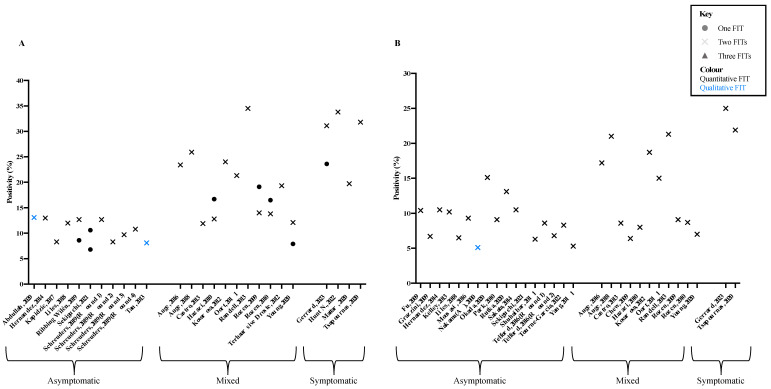
Positivity rates at (**A**) 10 µg Hb/g and (**B**) 20 µg Hb/g of the included studies. Positivity rates at a given threshold of one FIT, two FITs, and three FITs. Test was considered positive if any FIT was at or above the given threshold. Quantitative or qualitative FIT type is also shown [7,8,17,23,24,26,27,28,31,32,33,38,40,41,42,43,44,46,47,48,51,54,57,58,59,60,63,64,66,67,69,70,71,76,79,81].

The positivity rate at 10 µg Hb/g for using two FITs was between 8.1 and 34.5% (Figure 2A). Unsurprisingly, higher positivity rates were observed in the symptomatic cohorts. The highest two-test positivity rate (34.5%) was seen in a mixed cohort; the population in this study did not have a high rate of CRC (0.2%) or ACRN (2.4%), but the study was the only to use the Hemo Techt NS Plus (Alfresa Pharma, Osaka, Japan) FIT collection kit and analysis, more commonly, studies used the OC sensor (Eiken Chemical Co., Tokyo, Japan) or HM-JACKarc (Minaris Medical Co., Ltd., Tokyo, Japan) (Supplementary File S6) [67]. Where data were available for one- and two-test positivity at a 10 µg Hb/g threshold, the overall positivity increased by 3.0–9.2 percentage points with the addition of the second test. Again, greater increases were seen in the symptomatic and mixed study populations compared with the asymptomatic populations. At a threshold of 20 µg Hb/g, two-test positivity ranged between 5.1% and 25.0%, with an increase of 1.3–6.7 percentage points between one- and two-test strategies (Figure 2B).

### 3.2. Use of Additional FITs Leads to a Reduction in Missed CRC and ACRN

Twenty-six studies were eligible for assessment of diagnostic performance, given that a quantifiable FIT was used and there was an investigation or registry follow-up of all participants, regardless of FIT result. Seven of these were asymptomatic screening studies [26,29,30,31,38,40,44], thirteen contained mixed populations [56,57,58,59,63,64,66,67,69,70,71,73,76], and six contained symptomatic cohorts [7,8,77,79,80,81].

In nine studies, the diagnostic performance of one and then multiple FITs for detecting CRC at a threshold of 10 µg Hb/g was reported (Table 2). Four of these diagnosed all cases with the first FIT [26,59,63,67]. In one study, the second FIT failed to identify any of the four CRCs missed by the first [64], but in four other studies, further CRC was diagnosed due to the additional FIT [7,44,70,81]. This included an asymptomatic study in which only two missed CRCs were identified [44]. A mixed population study identified four of five CRCs missed by test one, and the extra case was identified by a third test [70]. And there were two symptomatic studies where the missed CRC rate was halved by the second test [7] and one missed case from single testing was picked up [81]. Where the outcome of interest was ACRN, there was a relative reduction in missed pathology of between 5.9 and 100% across the cohorts (Table 2C). The study, which identified all the cases of ACRN with the second test only, contained a small number of cases, and when excluded, the range of relative reduction was 5.933.5% [67]. The use of three tests, compared to one, reduced the missed ACRN rate by a relative 33.4–46.7% [63,69,70].

Using a 20 µg Hb/g threshold (Table 2B,D), in two studies, CRCs missed by one test were not identified with the addition of a second test (one and five cases of CRC, respectively) [63,64]. However, five studies reported a relative reduction of between 25.0 and 100% of missed CRC cases compared to using only one test [7,38,44,70,81]. For ACRN, similar reductions as seen at 10 µg Hb/g were observed.

Overall, these results show that the use of multiple FITs can be used to detect cases of CRC and ACRN that would have been undetected by a single FIT strategy.

### 3.3. Diagnostic Performance of Two-FIT Strategy

**Figure 3:**
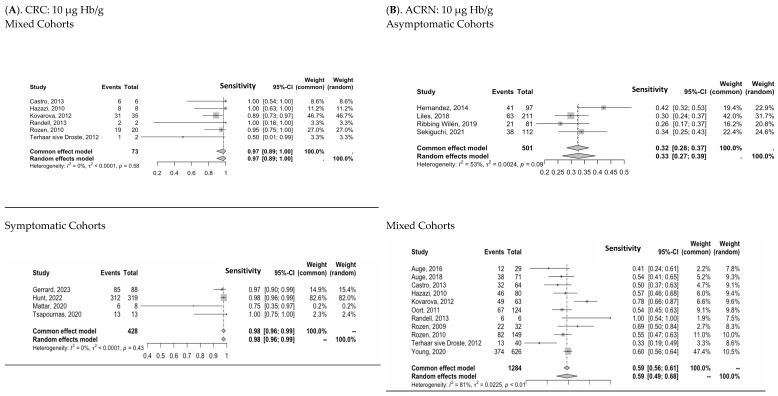

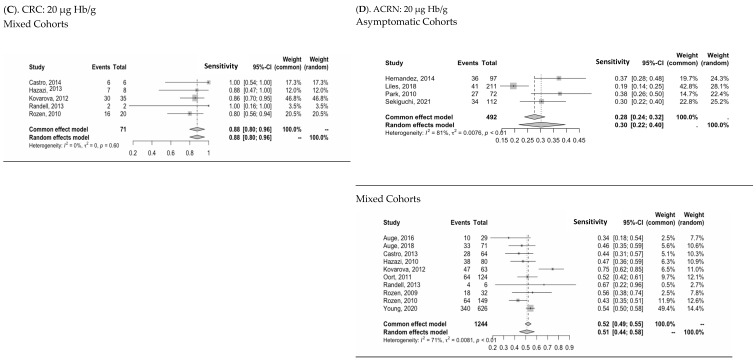
Meta-analysis for sensitivity of detecting CRC and ACRN at 10 µg Hb/g and 20 µg Hb/g for different study populations. [7,8,26,31,38,40,44,57,58,59,63,64,66,67,69,70,71,76,79,81].

A meta-analysis was performed to estimate the overall sensitivity for detecting CRC and ACRN using a double-FIT strategy at reported thresholds of 10 µg Hb/g and 20 µg Hb/g. The sensitivity for using two tests in mixed and symptomatic populations for diagnosing CRC at 10 µg Hb/g was 94% (95% CI: 88–100%) and 98% (95% CI: 96–99%), respectively (Figure 3). When the threshold was increased to 20 µg Hb/g, the sensitivity in the mixed cohort dropped to 88% (95% CI: 80–96%). For ACRN at 10 µg Hb/g, the sensitivity for asymptomatic cohorts was 33% (95% CI: 26–39%) and 59% (95% CI: 49–68%) in a mixed population. An increase in the threshold to 20 µg Hb/g led to a respective drop in sensitivity to 30% (95% CI: 21–39%) and 51% (95% CI: 44–58%) in the asymptomatic and mixed cohorts. These results highlight the high sensitivity for CRC using multiple FITs at a threshold of 10 µg Hb/g. The subsequent false positive rates are shown in Supplementary File S7.

## 4. Discussion

We have completed a systematic review and analysis of data from 68 studies reporting the use of more than one FIT in asymptomatic, mixed, and symptomatic populations. Analysis has shown that the use of a second test at 10 µg Hb/g increases the positivity by around 4.5 percentage points. Comparing additional CRC diagnoses made by the second test, four of five studies with cancer not diagnosed by test one found that at least half of the missed cancers were picked up through the second test. All studies showed an improvement in ACRN diagnosis with a median relative reduction in missed cases of 12.7%. This reduction was greatest in the mixed and symptomatic populations. Overall sensitivity for a double-FIT strategy for diagnosing CRC in mixed and symptomatic populations at 10 µg Hb/g was 94% and 98%, respectively.

Studies were appraised by the QUADAS-2 tool. The risk of bias was low in the studies included, as the screening population represented a random sample of the population, and the symptomatic patients were symptomatic of suspected lower GI pathology. The degree of bias from differing reference tests of colonoscopy, radiology, or no investigation for negative FIT varied accordingly. Bias in the FIT tests and in the histological results, following a colonoscopy, was minimal.

Within the four studies that reported increased CRC detection with a second test at the 10 µg Hb/g threshold, three reported first- and two-test positivity rates, with an observed increase of between 3.8 and 7.5 percentage points [7,44,81]. This increased workload, offset by an over 50% reduction in missed CRC, would support the use of a multiple FIT strategy particularly when being employed as a rule-out test and in high-risk populations. A further symptomatic study, not included in this review due to its methodology of not systematically retesting the included population but rather analysing cases where more than one test happened to have been performed, further supports the benefit of multiple testings [85]. In this analysis of repeated FITs (*n* = 5761) within a 12-month period, two negative FITs (<10 µg Hb/g) were associated with a very low rate of CRC (0.8%).

Our study builds on the descriptive systematic review article on repeated FITs in symptomatic patients from Farkas et al. [6]. In this study, we have included studies with asymptomatic and mixed populations along with more recent publications in large symptomatic cohorts. These additional studies have allowed for a more in-depth assessment and meta-analysis to be performed to answer key clinical questions. Previous analysis of single-FIT studies estimated the sensitivity for CRC diagnosis between 89 and 90% in symptomatic populations at 10 µg Hb/g [3,4,5]. Through a meta-analysis of double-FIT strategies, we estimate that two tests give a sensitivity of 98% (95% CI: 96–99%)

Whilst included initially for the assessment of test positivity, qualitative FIT studies and those without complete investigation were excluded from further analysis. This was an attempt to standardise, as much as possible, the evaluation of double-test sensitivity at given thresholds and in particular with regards to studies with unknown true false negative rates within their populations. This study is limited by its inclusion of only articles published in the English language. There were not enough studies with asymptomatic populations reporting two-test results for CRC to perform a meta-analysis. The meta-analysis for symptomatic studies contained only four studies, but this is the available literature. Combining and comparing results from different FIT analyses and study populations is challenging [86,87]. To best address this, studies were categorised based on the characteristics of the study population, and only quantitative FITs were taken forward for further analysis. The use of multiple FITs by way of a single FIT in multiple screening rounds was outside the scope of this paper but has been reviewed recently [88].

FIT is a relatively inexpensive, non-invasive test that is acceptable to the vast majority of participants [89]. It is a very good indicator for identifying patients with significant bowel pathology but is not perfect.

## 5. Conclusions

There appears to be a clinical benefit in reducing missed cases of CRC and ACRN by double-FIT to achieve a moderate increase in positivity. Particularly when FIT is used as a rule-out test or gatekeeper to further investigation, a two-test strategy can be beneficial. Further review studies utilising cost-effective analysis would be useful when evaluating the benefits of multiple FIT strategies.

## Figures and Tables

**Table 1:** Summary of included studies and their populations.

Author	Population
**Asymptomatic**	
Abdullah, 2020 [17]	Asymptomatic, Age 35–65
Cai, 2016 [18]	Asymptomatic, Age 40–74
Chubak, 2013 [19]	Asymptomatic, Age 50–74
Cole, 2003 [20]	Asymptomatic, Age 50–69
Dancourt, 2008 [21]	Asymptomatic, Age 50–74
Faivre, 2012 [22]	Asymptomatic, Age 50–74
Fu, 2009 [23]	Asymptomatic, Age 40 and Over
Grazzini, 2009 [24]	Asymptomatic, Age 50–69
Guittet, 2009 [25]	Asymptomatic, Age 50–74
Hernandez, 2014 [26]	Asymptomatic, Age 50–69
Kapidzic, 2017 [27]	Asymptomatic, Age 50–74
Kelley, 2013 [28]	Asymptomatic, Age 50–75
Launoy, 2005 [29]	Asymptomatic, Age 50–74
Levi, 2011 [30]	Asymptomatic, Age 50–75
Liles, 2018 [31]	Asymptomatic, Age 49–75
Moosavi, 2016 [32]	Asymptomatic, Age 50–75
Nakama (A), 2000 [33]	Asymptomatic, Age 40 and Over
Nakama (B), 2000 [34]	Asymptomatic, Age 40–60
Nakama, 2002 [35]	Asymptomatic, Adult Population
Nakazato, 2006 [36]	Asymptomatic, Adult Population
Okada, 2020 [37]	Asymptomatic, Age 50–75
Park, 2010 [38]	Asymptomatic, Age 50–74
Raginel, 2013 [39]	Asymptomatic, Age 50–74
Ribbing Wilén, 2019 [40]	Asymptomatic, Age 60
Rutka, 2020 [41]	Asymptomatic, Age 50–70
Sakata, 2014 [42]	Asymptomatic, Age 40 and Over
Schreuders, 2019 [43]	Asymptomatic, Age 50–74
Sekiguchi, 2021 [44]	Asymptomatic, Age 49–79
Shapiro, 2017 [45]	Asymptomatic, Age 50–74
Shuhaibar, 2011 [46]	Asymptomatic, Age 50 and Over
Smith, 2006 [82]	Asymptomatic, Age 50–75
Tan, 2013 [47]	Asymptomatic, Age 50 and Over
Telford, 2016 [48]	Asymptomatic, Age 50–74
Tepeš, 2014 [49]	Asymptomatic, Age 64–68
Tepeš, 2022 [50]	Asymptomatic, Age 50–74
Tourne-Garcia, 2022 [51]	Asymptomatic, Age 50–69
Wang, 2022 [52]	Asymptomatic, Age 40–74
Wong M.C.S, 2015 [53]	Asymptomatic, Age 50–74
Yang, 2011 [54]	Asymptomatic, Mean Age 55.18 ± 15.67
Ye, 2017 [55]	Asymptomatic, Age 40–74
**Mixed**	
Auge, 2013 [56]	Surveillance or Symptomatic, Adult Population
Auge, 2016 [57]	Surveillance or Symptomatic, Adult Population
Auge, 2018 [58]	Surveillance or Symptomatic, Adult Population
Castro, 2013 [59]	High Risk Screening of FDR with CRC, Adult Population
Chew, 2009 [60]	Screening, Surveillance or Symptomatic, Adult Population
Cruz-Correa, 2007 [61]	Screening, Surveillance, Previous CRC or Significant Family History, Adult Population
Guimarães, 2019 [62]	Screening, Surveillance or Symptomatic, Adult Population
Hazazi, 2010 [63]	High Risk Screening or Surveillance, Adult Population
Kovarova, 2012 [64]	Screening, Surveillance or Symptomatic, Adult Population
Li, 2006 [65]	Screening, Surveillance or Symptomatic, Adult Population
Oort, 2011 [66]	Screening, Surveillance or Symptomatic, Adult Population
Randell, 2013 [67]	High Risk Screening or Symptomatic, Adult Population
Redwood, 2014 [68]	Asymptomatic or Surveillance, Adult Population
Rozen, 2009 [69]	High Risk Screening, Surveillance or Symptomatic, Adult Population
Rozen, 2010 [70]	High Risk Screening, Surveillance or Symptomatic, Adult Population
Terhaar sive Droste, 2012 [71]	High Risk Screening or Surveillance, Adult Population
Vasilyev, 2015 [72]	Screening, Surveillance or Symptomatic, Adult Population
Wong B.C, 2003 [73]	Surveillance or Symptomatic, Adult Population
Wong W.M, 2003 [74]	Surveillance or Symptomatic, Adult Population
Wu, 2014 [75]	Screening, Surveillance or Symptomatic, Adult Population
Young, 2020 [76]	High Risk Screening or Surveillance, Adult Population
**Symptomatic**	
Fernández-Bañares, 2019 [77]	Symptomatic, Adult Population
Gerrard, 2023 [7]	Symptomatic, Adult Population
Högberg, 2020 [78]	Symptomatic, Adult Population
Hunt N., 2022 [8]	Symptomatic, Adult Population
Mattar, 2020 [79]	Symptomatic, Adult Population
Oono, 2010 [80]	Symptomatic, Adult Population
Smith, 2006 [82]	Symptomatic, Adult Population
Tsapournas, 2020 [81]	Symptomatic, Adult Population

**Table 2:** Reduction in missed CRC and ACRN between one and multiple FITs at thresholds of 10 µg Hb/g and 20 µg Hb/g.

Population	Author	CRCs	1T FN	2T FN	3T FN	Relative Reduction in Missed CRC by 2T	Relative Reduction in Missed CRC by 3T
**A. 10 µg Hb/g: CRC**							
Asymptomatic	Hernandez, 2014 [26]	5	0	0	-	n/a	-
	Sekiguchi, 2021 [44]	10	2	0	-	100%	-
Mixed	Castro, 2014 [59]	6	0	0	-	n/a	-
	Hazazi, 2010 [63]	8	0	0	0	n/a	n/a
	Kovarova, 2012 [64]	35	4	4	-	0.0%	-
	Randell, 2013 [67]	2	0	0	-	n/a	-
	Rozen, 2010 [70]	20	5	1	0	80.0%	100%
Symptomatic	Gerrard, 2023 [7]	88	6	3	-	50.0%	-
	Tsapournas, 2020 [81]	13	1	0	-	100%	-
**B. 20 µg Hb/g: CRC**							
Asymptomatic	Hernandez, 2014 [26]	5	0	0	-	n/a	-
	Park, 2010 [38]	13	4	2	2	50.0%	50.0%
	Sekiguchi, 2021 [44]	10	2	0	-	100%	-
Mixed	Castro, 2014 [59]	6	0	0	-	n/a	-
	Hazazi, 2010 [63]	8	1	1	0	0.0%	100%
	Kovarova, 2012 [64]	35	5	5	-	0.0%	-
	Randell, 2013 [67]	2	0	0	-	n/a	-
	Rozen, 2010 [70]	20	7	4	5	42.9%	71.4%
Symptomatic	Gerrard, 2023 [7]	88	12	9	-	25.0%	-
	Tsapournas, 2020 [81]	13	2	1	-	50.0%	-
**Population**	**Author**	**ACRNs**	**1T FN**	**2T FN**	**3T FN**	**Relative Reduction in Missed ACRN by 2T**	**Relative Reduction in Missed ACRN by 3T**
**C. 10 µg Hb/g: ACRN**							
Asymptomatic	Hernandez, 2014 [26]	97	63	56	-	11.1%	-
	Liles, 2018 [31]	211	163	148	-	9.2%	-
	Ribbing Wilén, 2019 [40]	81	65	60	-	7.7%	-
	Sekiguchi, 2021 [44]	112	85	74	-	12.9%	-
Mixed	Auge, 2016 [57]	29	19	17		10.5%	-
	Auge, 2018 [58]	71	39	33	-	13.3%	
	Castro, 2014 [59]	64	34	32	-	5.9%	-
	Hazazi, 2010 [63]	80	44	34	28	22.5%	37.6%
	Kovarova, 2012 [64]	63	15	14	-	6.7%	-
	Oort, 2011 [66]	124	65	57	-	12.3%	-
	Randell, 2013 [67]	6	1	0	-	100%	-
	Rozen, 2009 [69]	32	15	10	8	33.5%	46.7%
	Rozen, 2010 [70]	149	87	67	58	22.9%	33.4%
	Young, 2020 [76]	626	350	252	-	28.0%	-
Symptomatic	Gerrard, 2023 [7]	185	48	34	-	29.2%	-
	Tsapournas, 2020 [81]	28	8	7	-	12.5%	-
**D. 20 µg Hb/g: ACRN**							
Asymptomatic	Hernandez, 2014 [26]	97	66	61	-	7.6%	-
	Liles, 2018 [31]	211	181	170	-	5.9%	-
	Park, 2010 [38]	72	49	45	41	9.0%	17.3%
	Sekiguchi, 2021 [44]	112	87	78	-	10.4%	
Mixed	Auge, 2016 [57]	29	20	19	-	5.1%	-
	Auge, 2018 [58]	71	45	38	-	15.9%	-
	Castro, 2014 [59]	64	38	36	-	5.3%	-
	Hazazi, 2010 [63]	80	52	42	32	19.1%	38.3%
	Kovarova, 2012 [64]	63	18	16	-	11.2%	-
	Oort, 2011 [66]	124	68	60	-	11.8%	-
	Randell, 2013 [67]	6	2	2	-	0.0%	-
	Rozen, 2009 [69]	32	17	14	10	17.7%	41.2%
	Rozen, 2010 [70]	149	102	85	73	16.8%	28.5%
	Young, 2020 [76]	626	413	286	-	30.8%	-
Symptomatic	Gerrard, 2023 [7]	185	64	46	-	28.1%	-
	Tsapournas, 2020 [81]	28	11	9	-	18.2%	-

CRC: colorectal cancer; ACRN: advanced colorectal neoplasia; 1T: one (first) FIT; 2T: two-test FIT (any positive); 3T: three-test FIT (any positive); FN: false negative.

## Data Availability

Summarised anonymised data will be made available on request.

## Supplementary Materials

The following supporting information can be downloaded at https://www.mdpi.com/article/10.3390/cancers16183199/s1. Supplementary File S1: Search Strategies in (A) MEDLINE, (B) EMBASE and (C) Cochrane Central Register of Controlled Trials; Supplementary File S2: QUADAS-2 appraisal of risk of bias; Supplementary File S3: Funnel plots and egger test for performed Meta-analysis; Supplementary File S4: Studies to use multiple FIT but not meet inclusion criteria; Supplementary File S5: Summary of included studies; Supplementary File S6: (A) FIT positivity by number of tests and thresholds utilised. (B) Two test positivity at 10µg by FIT analyser; Supplementary File S7: False positive rates at differing thresholds and populations for (A) CRC and (B) ACRN.
