# Supplementary material for: Repeat Faecal Immunochemical Testing for Colorectal Cancer Detection in Symptomatic and Screening Patients: A Systematic Review and Meta-Analysis
Source: Cancers (Basel). 2024 Sep 19;16(18):3199. doi: 10.3390/cancers16183199 (PMC11429846; doi:10.3390/cancers16183199)
Supplement: Supplementary file 1 [file cancers-16-03199-s001.zip › cancers-3166488-supplementary.pdf]

Title:

Repeat Faecal Immunochemical Testing for Colorectal Cancer Detection in Symptomatic and Screening Patients: A Systematic Review and Meta-Analysis

Authors:

Adam D. Gerrard [1,2], Roberta Garau [1,2], Wei Xu [1,3], Yasuko Maeda [4,5], Malcolm G. Dunlop [1,6], Evropi Theodoratou [1,3] and Farhat V. N. Din [1,2,*]

1 Cancer Research UK Scotland Centre, Institute of Genetics and Cancer, University of Edinburgh, Edinburgh EH4 2XR, UK; adam.gerrard@ed.ac.uk (A.D.G.); e.theodoratou@ed.ac.uk (E.T.)
2 Department of Colorectal Surgery, Western General Hospital, Edinburgh EH4 2XU, UK
3 Centre for Global Health, Usher Institute, The University of Edinburgh, Edinburgh EH4 2XR, UK
4 School of Medicine, Dentistry and Nursing, University of Glasgow, Glasgow G12 8QQ, UK
5 Department of Surgery, Queen Elizabeth University Hospital, Glasgow G51 4TF, UK
6 UK Colon Cancer Genetics Group, Medical Research Council Human Genetics Unit, Medical Research Council Institute of Genetics & Cancer, Western General Hospital, The University of Edinburgh, Edinburgh EH4 2XU, UK
* Correspondence: farhat.din@ed.ac.uk

Supplementary Materials Index

File S1:
Search Strategies in (A) MEDLINE, (B) EMBASE and
(C) Cochrane Central Register of Controlled Trials pg. 2

File S2:
QUADAS-2 appraisal of risk of bias pg.3

File S3: pg.4-7
Funnel plots and egger test for performed Meta-analysis

File S4: pg.8-12
Studies to use multiple FIT but not meet inclusion criteria

File S5: pg.13
Summary of included studies

File S6: pg.14-18
A) FIT positivity by number of tests and thresholds utilised.
(B) Two test positivity at 10µg by FIT analyser

File S7: pg.19
False positive rates

Supplementary File S1: Search Strategies in (A) MEDLINE, (B) EMBASE and (C) Cochrane Central Register of Controlled Trials

A. MEDLINE

▼ **Search History** (13) View Saved

# ▲	Searches	Results	Type	Actions	Annotations
1	(colon* or colorectal or rectum or rectal).mp. [mp=title, book title, abstract, original title, name of substance word, subject heading word, floating sub-heading word, keyword heading word, organism supplementary concept word, protocol supplementary concept word, rare disease supplementary concept word, unique identifier, synonyms, population supplementary concept word, anatomy supplementary concept word]	790572	Advanced	Display Results More	
2	exp Colorectal Neoplasms/	235225	Advanced	Display Results More	
3	exp Rectal Neoplasms/	53619	Advanced	Display Results More	
4	exp Colonic Neoplasms/	82056	Advanced	Display Results More	
5	2 or 3 or 4	235225	Advanced	Display Results More	
6	exp Colonic Polyps/ or exp Polyps/ or exp Adenomatous Polyps/ or exp Intestinal Polyps/	42058	Advanced	Display Results More	
7	5 or 6	259327	Advanced	Display Results More	
8	1 and 7	232357	Advanced	Display Results More	
9	(immunochemi* adj3 (stool* or fec* or faec*)).mp. [mp=title, book title, abstract, original title, name of substance word, subject heading word, floating sub-heading word, keyword heading word, organism supplementary concept word, protocol supplementary concept word, rare disease supplementary concept word, unique identifier, synonyms, population supplementary concept word, anatomy supplementary concept word]	1855	Advanced	Display Results More	
10	(iFOBT or FOBT or iFIT).mp. [mp=title, book title, abstract, original title, name of substance word, subject heading word, floating sub-heading word, keyword heading word, organism supplementary concept word, protocol supplementary concept word, rare disease supplementary concept word, unique identifier, synonyms, population supplementary concept word, anatomy supplementary concept word]	1591	Advanced	Display Results More	
11	9 or 10	3181	Advanced	Display Results More	
12	8 and 11	2818	Advanced	Display Results More	
13	limit 12 to yr="2000 - 2022"	2622	Advanced	Display Results More	

Save Remove Combine with: AND OR

B. EMBASE

▼ **Search History** (16) View Saved

# ▲	Searches	Results	Type	Actions	Annotations
1	(colon* or colorectal or rectal or rectal).mp. [mp=title, abstract, heading word, drug trade name, original title, device manufacturer, drug manufacturer, device trade name, keyword heading word, floating subheading word, candidate term word]	1355077	Advanced	Display Results More	
2	exp colorectal tumor/	454102	Advanced	Display Results More	
3	exp rectum tumor/	76246	Advanced	Display Results More	
4	exp Rectal Neoplasms/	76246	Advanced	Display Results More	
5	exp colorectal tumor/	454102	Advanced	Display Results More	
6	exp colorectal cancer/	382616	Advanced	Display Results More	
7	exp Colonic Neoplasms/	168088	Advanced	Display Results More	
8	2 or 3 or 4 or 5 or 6 or 7	454102	Advanced	Display Results More	
9	exp colon polyp/ or exp polyp/ or exp adenomatous polyp/ or exp intestine polyp/ or exp rectum polyp/ or exp colorectal polyp/	88277	Advanced	Display Results More	
10	8 or 9	502889	Advanced	Display Results More	
11	1 and 10	454255	Advanced	Display Results More	
12	(immunochemi* adj3 (stool* or fec* or faec*)).mp. [mp=title, abstract, heading word, drug trade name, original title, device manufacturer, drug manufacturer, device trade name, keyword heading word, floating subheading word, candidate term word]	4072	Advanced	Display Results More	
13	(iFOBT or iFIT or FOBT).mp. [mp=title, abstract, heading word, drug trade name, original title, device manufacturer, drug manufacturer, device trade name, keyword heading word, floating subheading word, candidate term word]	3437	Advanced	Display Results More	
14	12 or 13	6960	Advanced	Display Results More	
15	11 and 14	5870	Advanced	Display Results More	
16	limit 15 to yr="2000 - 2022"	5597	Advanced	Display Results More	

Save Remove Combine with: AND OR

C. Cochrane Central Register of Controlled Trials

View fewer lines | Print search history

#	Search			Results
#1	(Colon*):ti,ab,kw OR (Colorectal):ti,ab,kw OR (rectal):ti,ab,kw OR (rectum):ti,ab,kw	S	Limits	64093
#2	(colorectal tumour):ti,ab,kw OR (colorectal tumor):ti,ab,kw OR ("colorectal cancer"):ti,ab,kw OR (colorectal neoplasm):ti,ab,kw	S	Limits	16588
#3	(rectal tumour):ti,ab,kw OR (rectal tumor):ti,ab,kw OR ("rectal cancer"):ti,ab,kw OR ("rectal neoplasm"):ti,ab,kw	S	Limits	4610
#4	(colon tumour):ti,ab,kw OR (colon tumor):ti,ab,kw OR (colon cancer):ti,ab,kw OR (colon neoplasm):ti,ab,kw	S	Limits	7166
#5	#2 OR #3 Or #4		Limits	23898
#6	(colon polyp):ti,ab,kw OR (bowel polyp):ti,ab,kw OR ("polyp):ti,ab,kw OR ("intestinal polyposis"):ti,ab,kw	S	Limits	3654
#7	#5 OR #6		Limits	26447
#8	#1 AND #7		Limits	25127
#9	(immunochemi*):ti,ab,kw	S	Limits	698
#10	(FIT):ti,ab,kw OR (FOBT):ti,ab,kw OR (faecal immunochemical*):ti,ab,kw OR (fecal immunochemical*):ti,ab,kw	S	Limits	15596
#11	#9 OR #10		Limits	15771
#12	#8 AND #11		Limits	1088

✖ Clear all

☐ Highlight orphan lines

Supplementary File S2: QUADAS-2 appraisal of risk of bias

Study	RISK OF BIAS				APPLICABILITY CONCERNS		
	PATIENT SELECTION	INDEX TEST	REFERENCE STANDARD	FLOW AND TIMING	PATIENT SELECTION	INDEX TEST	REFERENCE STANDARD
**Asymptomatic**							
Abdullah, 2020	Low Risk	Low Risk	Unclear Risk	Low Risk	Low Risk	Low Risk	Unclear Risk
Cai, 2016	Low Risk	Low Risk	Unclear Risk	Low Risk	Low Risk	Low Risk	Unclear Risk
Chubak, 2013	Low Risk	Low Risk	Unclear Risk	Low Risk	Low Risk	Low Risk	Unclear Risk
Cole, 2003	Low Risk	Low Risk	Unclear Risk	Low Risk	Low Risk	Low Risk	Unclear Risk
Dancourt, 2008	Low Risk	Low Risk	Unclear Risk	Low Risk	Low Risk	Low Risk	Unclear Risk
Faivre, 2012	Low Risk	Low Risk	Unclear Risk	Low Risk	Low Risk	Low Risk	Unclear Risk
Fu, 2009	Unclear Risk	Low Risk	Unclear Risk	Low Risk	Unclear Risk	Low Risk	Unclear Risk
Grazzini, 2009	Low Risk	Low Risk	Unclear Risk	Low Risk	Low Risk	Low Risk	Unclear Risk
Guittet, 2009	Low Risk	Low Risk	Unclear Risk	Low Risk	Low Risk	Low Risk	Unclear Risk
Hernandez, 2014	Low Risk	Low Risk	Low Risk	Low Risk	Low Risk	Low Risk	Low Risk
Kapidzic, 2017	Low Risk	Low Risk	Unclear Risk	Low Risk	Low Risk	Low Risk	Unclear Risk
Kelley, 2013	Low Risk	Low Risk	Unclear Risk	Low Risk	Low Risk	Low Risk	Unclear Risk
Launoy, 2005	Low Risk	Low Risk	Low Risk	Low Risk	Low Risk	Low Risk	Low Risk
Levi, 2011	Low Risk	Low Risk	Unclear Risk	Low Risk	Low Risk	Low Risk	Unclear Risk
Liles, 2018	Low Risk	Low Risk	Low Risk	Low Risk	Low Risk	Low Risk	Low Risk
Moosavi, 2016	Low Risk	Low Risk	Unclear Risk	Low Risk	Low Risk	Low Risk	Unclear Risk
Nakama (A), 2000	Low Risk	Low Risk	Low Risk	Low Risk	Low Risk	Low Risk	Low Risk
Nakama (B), 2000	Low Risk	Low Risk	Low Risk	Low Risk	Low Risk	Low Risk	Low Risk
Nakama, 2002	Unclear Risk	Low Risk	Low Risk	Low Risk	Unclear Risk	Low Risk	Low Risk
Nakazato, 2006	Low Risk	Low Risk	Unclear Risk	Low Risk	Low Risk	Low Risk	Unclear Risk
Okada, 2020	Low Risk	Low Risk	Unclear Risk	Low Risk	Low Risk	Low Risk	Unclear Risk
Park, 2010	Low Risk	Low Risk	Low Risk	Low Risk	Low Risk	Low Risk	Low Risk
Raginel, 2013	Low Risk	Low Risk	Unclear Risk	Low Risk	Low Risk	Low Risk	Unclear Risk
Ribbing Wilén, 2019	Low Risk	Low Risk	Low Risk	Low Risk	Low Risk	Low Risk	Low Risk
Rutka, 2020	Low Risk	Low Risk	Unclear Risk	Low Risk	Low Risk	Low Risk	Unclear Risk
Sakata, 2014	Low Risk	Low Risk	Unclear Risk	Low Risk	Low Risk	Low Risk	Unclear Risk
Schreuders, 2019	Low Risk	Low Risk	Unclear Risk	Low Risk	Low Risk	Low Risk	Unclear Risk
Sekiguchi, 2021	Low Risk	Low Risk	Low Risk	Low Risk	Low Risk	Low Risk	Low Risk
Shapiro, 2017	Low Risk	Low Risk	Low Risk	Low Risk	Low Risk	Low Risk	Low Risk
Shuhaibar, 2011	Unclear Risk	Low Risk	Unclear Risk	Low Risk	Unclear Risk	Low Risk	Unclear Risk
Tan, 2013	Low Risk	Low Risk	Unclear Risk	Low Risk	Low Risk	Low Risk	Unclear Risk
Telford, 2016	Low Risk	Low Risk	Unclear Risk	Low Risk	Low Risk	Low Risk	Unclear Risk
Tepeš, 2014	Low Risk	Low Risk	Unclear Risk	Low Risk	Low Risk	Low Risk	Unclear Risk
Tepeš, 2022	Low Risk	Low Risk	Unclear Risk	Low Risk	Low Risk	Low Risk	Unclear Risk
Tourne-Garcia, 2022	Low Risk	Low Risk	Unclear Risk	Low Risk	Low Risk	Low Risk	Unclear Risk
Wang, 2022	Low Risk	Low Risk	Unclear Risk	Low Risk	Low Risk	Low Risk	Unclear Risk
Wong M.C.S, 2015	Low Risk	Low Risk	Low Risk	Low Risk	Low Risk	Low Risk	Low Risk
Yang, 2011	Low Risk	Low Risk	Unclear Risk	Low Risk	Low Risk	Low Risk	Unclear Risk
Ye, 2017	Low Risk	Low Risk	Unclear Risk	Low Risk	Low Risk	Low Risk	Unclear Risk
**Mixed**							
Auge, 2013	Low Risk	Low Risk	Low Risk	Low Risk	Low Risk	Low Risk	Low Risk
Auge, 2016	Low Risk	Low Risk	Low Risk	Low Risk	Low Risk	Low Risk	Low Risk
Auge, 2018	Low Risk	Low Risk	Low Risk	Low Risk	Low Risk	Low Risk	Low Risk
Castro, 2013	Low Risk	Low Risk	Low Risk	Low Risk	Low Risk	Low Risk	Low Risk
Chew, 2009	Unclear Risk	Low Risk	Unclear Risk	Low Risk	Unclear Risk	Low Risk	Unclear Risk
Cruz-Correa, 2007	Low Risk	Low Risk	Low Risk	Low Risk	Low Risk	Low Risk	Low Risk
Guimarães, 2019	Low Risk	Low Risk	Low Risk	Low Risk	Low Risk	Low Risk	Low Risk
Hazazi, 2010	Unclear Risk	Low Risk	Low Risk	Low Risk	Unclear Risk	Low Risk	Low Risk
Kovarova, 2012	Low Risk	Low Risk	Low Risk	Low Risk	Low Risk	Low Risk	Low Risk
Li, 2006	Low Risk	Low Risk	Low Risk	Low Risk	Low Risk	Low Risk	Low Risk
Oort, 2011	Low Risk	Low Risk	Low Risk	Low Risk	Low Risk	Low Risk	Low Risk
Randell, 2013	Low Risk	Low Risk	Low Risk	Low Risk	Low Risk	Low Risk	Low Risk
Redwood, 2014	Unclear Risk	Low Risk	Low Risk	Low Risk	Unclear Risk	Low Risk	Low Risk
Rozen, 2009	Unclear Risk	Low Risk	Low Risk	Low Risk	Unclear Risk	Low Risk	Low Risk
Rozen, 2010	Unclear Risk	Low Risk	Low Risk	Low Risk	Unclear Risk	Low Risk	Low Risk
Terhaar sive Droste, 2012	Low Risk	Low Risk	Low Risk	Low Risk	Low Risk	Low Risk	Low Risk
Vasilyev, 2015	Low Risk	Low Risk	Low Risk	Low Risk	Low Risk	Low Risk	Low Risk
Wong B.C, 2003	Low Risk	Low Risk	Low Risk	Low Risk	Low Risk	Low Risk	Low Risk
Wong W.M, 2003	Low Risk	Low Risk	Low Risk	Low Risk	Low Risk	Low Risk	Low Risk
Wu, 2014	Low Risk	Low Risk	Low Risk	Low Risk	Low Risk	Low Risk	Low Risk
Young, 2020	Low Risk	Low Risk	Low Risk	Low Risk	Low Risk	Low Risk	Low Risk
**Symptomatic**							
Fernández-Bañares, 2019	Low Risk	Low Risk	Low Risk	Low Risk	Low Risk	Low Risk	Low Risk
Gerrard, 2023	Low Risk	Low Risk	Unclear Risk	Low Risk	Low Risk	Low Risk	Unclear Risk
Högberg, 2020	Unclear Risk	Low Risk	Unclear Risk	Unclear Risk	Low Risk	Low Risk	Low Risk
Hunt N, 2022	Low Risk	Low Risk	Unclear Risk	Low Risk	Low Risk	Low Risk	Unclear Risk
Mattar, 2020	Low Risk	Low Risk	Low Risk	Low Risk	Low Risk	Low Risk	Low Risk
Oono, 2010	Low Risk	Low Risk	Low Risk	Low Risk	Low Risk	Low Risk	Low Risk
Smith , 2006	Low Risk	Low Risk	Low Risk	Low Risk	Low Risk	Low Risk	Low Risk
Tsapournas, 2020	Low Risk	Low Risk	Low Risk	Low Risk	Low Risk	Low Risk	Low Risk

Supplementary File S3: Funnel plots and egger test for performed Meta-analysis

Detection of CRC in mixed cohorts at a threshold of 10µg Hb/g

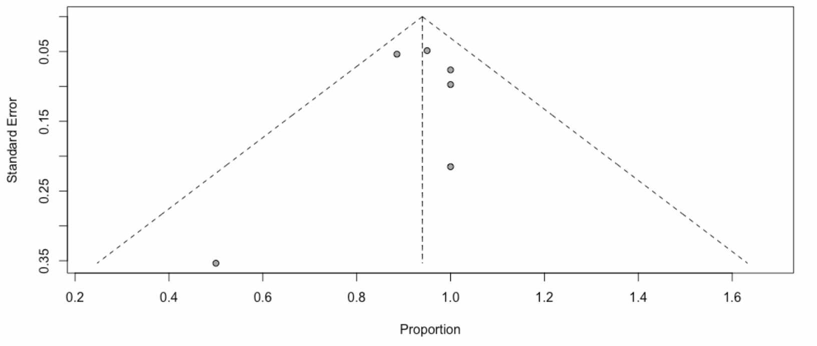

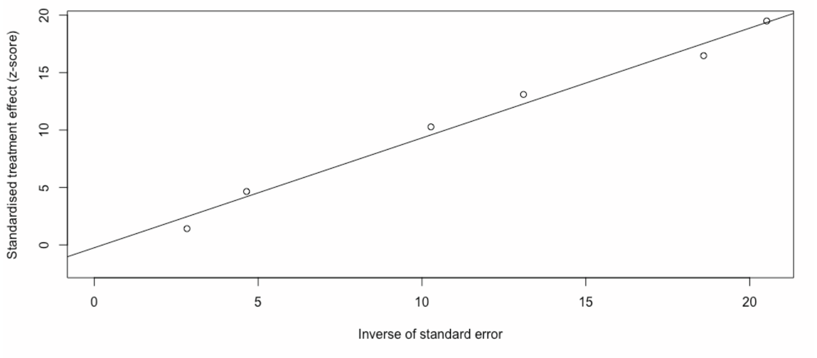

Detection of CRC in symptomatic cohorts at a threshold of 10µg Hb/g

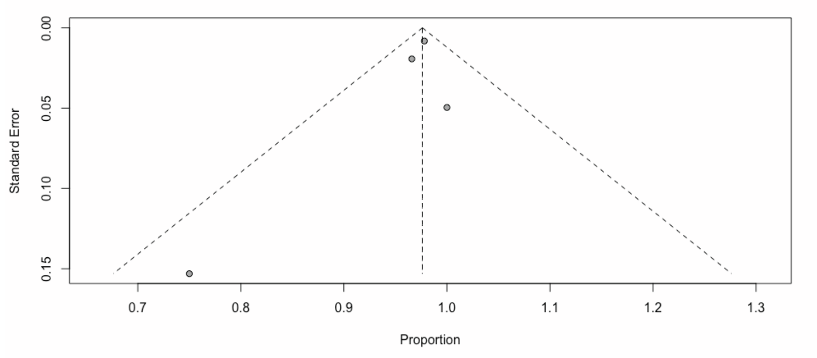

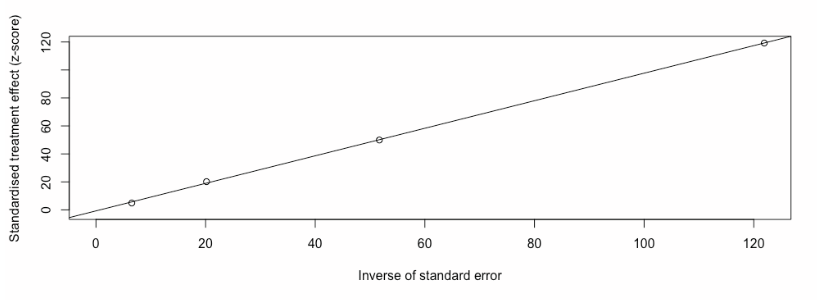

Detection of ACRN in asymptomatic cohorts at a threshold of 10µg Hb/g

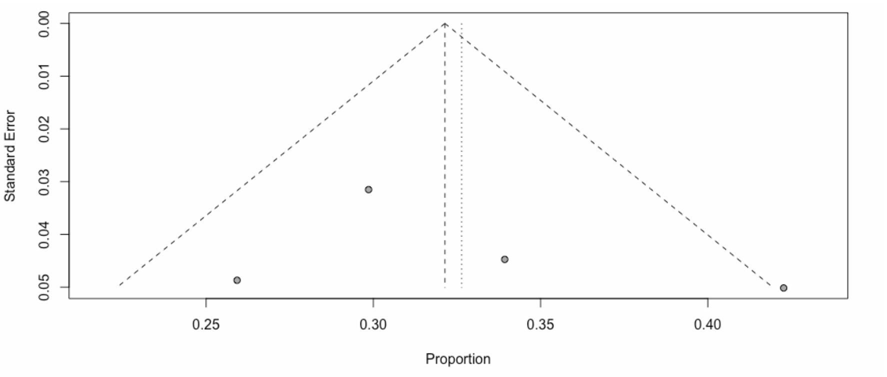

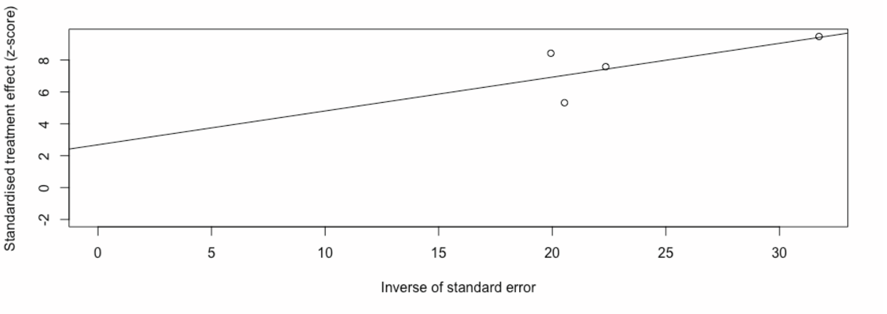

Detection of ACRN in mixed cohorts at a threshold of 10µg Hb/g

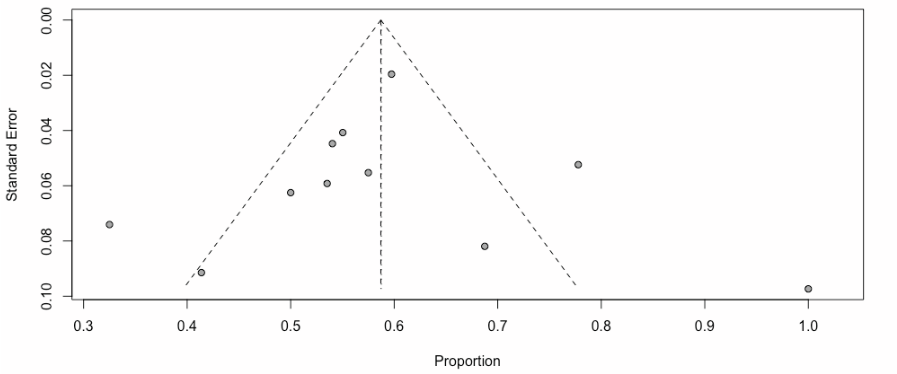

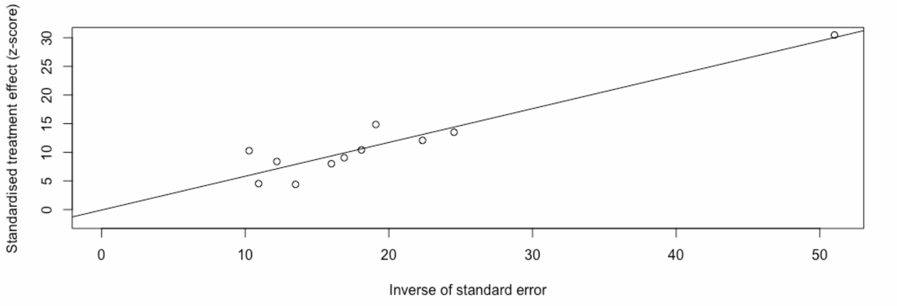

Detection of CRC in mixed cohorts at a threshold of 20µg Hb/g

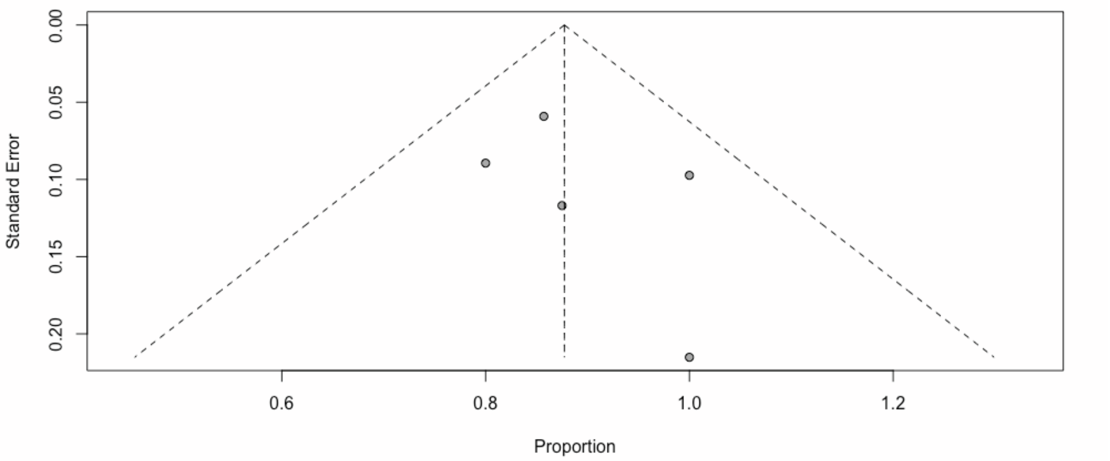

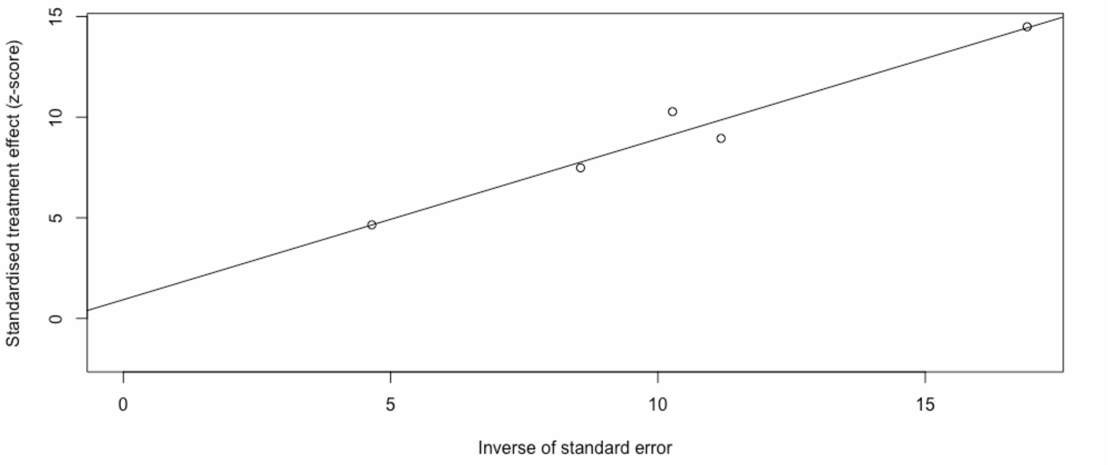

Detection of ACRN in asymptomatic cohorts at a threshold of 20µg Hb/g

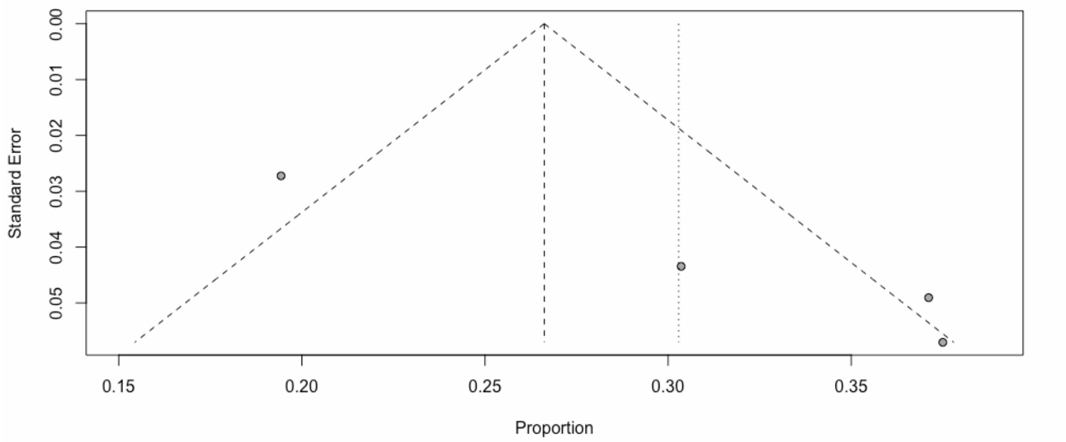

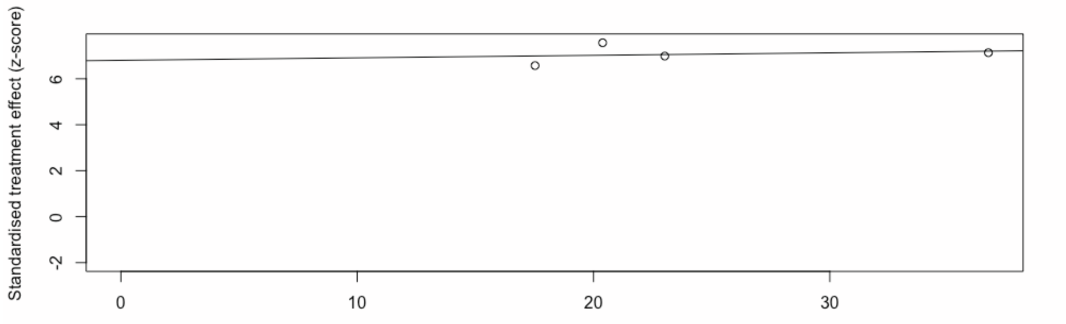

Detection of ACRN in mixed cohorts at a threshold of 20μg Hb/g

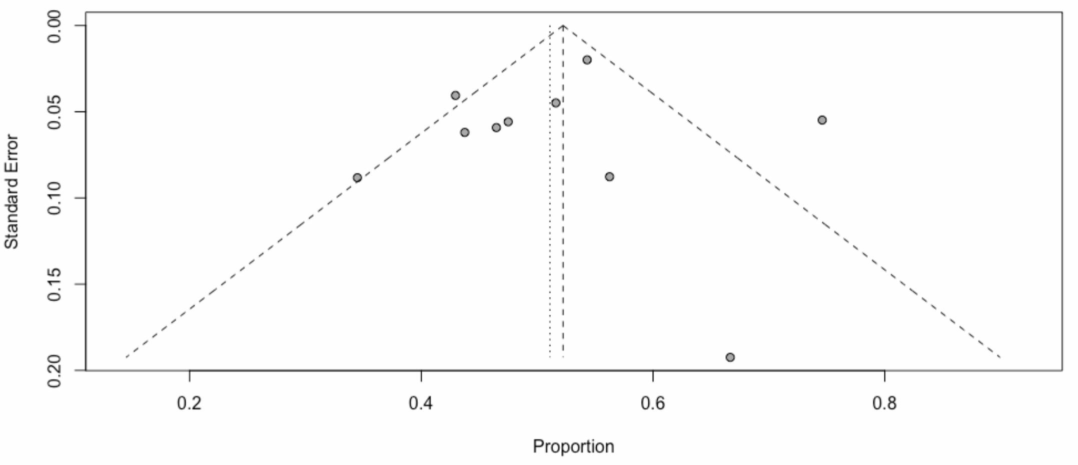

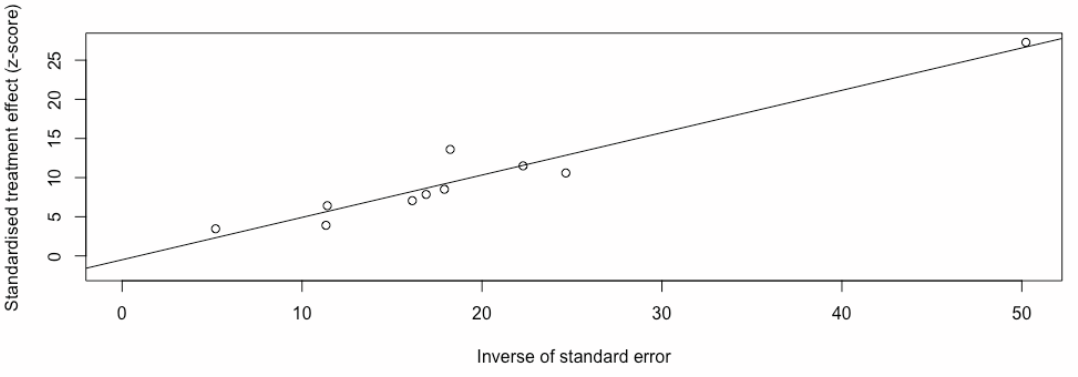

Supplementary File S4: Studies to use multiple FIT but not meet inclusion criteria

Author	Year	Title	Reason For Exclusion
Abu Hassan	2016	Evaluation of a Colorectal Carcinoma Screening Program in Kota Setar and Kuala Muda Districts, Malaysia	No details of FIT used
Amitay	2019	Fecal Immunochemical Tests for Colorectal Cancer Screening: Is Fecal Sampling from Multiple Sites Necessary?	Repeat samples from the same stool
Augue	2014	Risk Stratification for Advanced Colorectal Neoplasia According to FecalHemoglobin Concentration in a Colorectal Cancer Screening Program	No data for FIT diagnostic performance of CRC or ACRN at set thresholds
Benton	2022	A comparison of the faecal haemoglobin concentrations and diagnostic accuracy in patients suspected with colorectal cancer and serious bowel disease as reported on four different faecal immunochemical test systems	Single test of multiple FITs
Borao	2023	A Second FIT After A First Weak Positive One As A Useful Strategy To Reduce Colonoscopy Load In CRC Screening	Only weakly positive tests were repeated
Brenner	2013	Superior diagnostic performance of faecal immunochemical tests for haemoglobin in a head-to-head comparison with guaiac based faecal occult blood test among 2235 participants of screening colonoscopy	Single test of multiple FITs
Bujanda	2015	Colorectal cancer in a second round after a negative faecal immunochemical test	Single sampling over multiple screening rounds
Buron	2019	Changes in FIT values below the threshold of positivity and short-term risk of advanced colorectal neoplasia: Results from a population-based cancer screening program	Single sampling over multiple screening rounds
Cai	2011	Performance of a Colorectal Cancer Screening Protocol in an Economically and Medically Underserved Population	Data included in further study (Cai, 2016)
Cai	2011	Performance of a colorectal cancer screening protocol in an economically and medically underserved population.	Data included in further study (Cai, 2016)
Castiglione	2000	Screening for colorectal cancer by faecal occult blood test: comparison of immunochemical tests	Single test of multiple FITs
Chen H.	2020	Comparative Evaluation of Participation and Diagnostic Yield of Colonoscopy vs Fecal Immunochemical Test vs Risk-Adapted Screening in Colorectal Cancer Screening: Interim Analysis of a Multicenter Randomized Controlled Trial (TARGET-C)	Single sampling over multiple screening rounds
Chiang	2015	Timing and Risk Factors for a Positive Fecal Immunochemical Test in Subsequent Screening for Colorectal Neoplasms	Single sampling over multiple screening rounds
Davis	2020	Repeat Annual Colorectal Cancer Screening in Rural Community Clinics: A Randomized Clinical Trial to Evaluate Outreach Strategies to Sustain Screening	Single sampling over multiple screening rounds
de Klerk	2018	Performance of two faecal immunochemical tests for the detection of advanced neoplasia at different positivity thresholds: a cross-sectional study of the Dutch national colorectal cancer screening programme.	Single test of two FITs
Dube	2019	Number of samples in faecal immunochemical test screening: more might be less	Comment on Schreuders (2019)
Graser	2013	Comparison of CT colonography, colonoscopy, sigmoidoscopy and faecal occult blood tests for the detection of advanced adenoma in an average risk population.	Repeat samples from the same stool

Grobbee	2020	Diagnostic Yield of One-Time Colonoscopy vs One-Time Flexible Sigmoidoscopy vs Multiple Rounds of Mailed Fecal Immunohistochemical Tests in Colorectal Cancer Screening	Single sampling over multiple screening rounds
Guittet	2007	Comparison of a guaiac based and an immunochemical faecal occult blood test in screening for colorectal cancer in a general average risk population	Data included in further study (Guittet, 2009)
Guittet	2009	Comparison of a guaiac and an immunochemical faecal occult blood test for the detection of colonic lesions according to lesion type and location	Data included in further study (Guittet, 2009)
Guittet	2012	Colorectal cancer screening: why immunochemical faecal occult blood test performs as well with either one or two samples	Data included in further study (Guittet, 2009)
Hancock	2019	Detection Rate of Colorectal Cancer or Precancer Adenoma by Colonoscopy After 1, 2, or 3 Positive Results via Fecal Immunochemical Testing	Repeat samples from the same stool
Heisser	2020	Age-specific sequence of colorectal cancer screening options in Germany: A model- based critical evaluation	Single sampling over multiple screening rounds
Hiroshi	2011	Effectiveness of repeated screening using the fecal occult blood test and its impact on reducing false-negative cancer cases	Single sampling over multiple screening rounds
Högberg	2013	Immunochemical faecal occult blood tests in primary care and the risk of delay in the diagnosis of colorectal cancer	Retrospective review of CRCs and adenomas with HGD
Högberg	2017	Faecal immunochemical tests for the diagnosis of symptomatic colorectal cancer in primary care: the benefit of more than one sample	Retrospective review of CRCs and adenomas with HGD
Högberg	2020	Qualitative faecal immunochemical tests (FITs) for diagnosing colorectal cancer in patients with histories of rectal bleeding in primary care: a cohort study	Subset data from Högberg, 2020
Huang	2014	Predictive power of quantitative and qualitative fecal immunochemical tests for hemoglobin in population screening for colorectal neoplasm	Single test of two FITs
Hunt J.S	2022	A True Positive and a False Negative? The Dilemma of Negative Colonoscopy After a Positive Fecal Occult Blood Test	Followed up positive FITs with negative colonoscopy with a further colonoscopy
Johnstone	2022	Prevalence of repeat faecal immunochemical testing in symptomatic patients attending primary care	Repeat samples within 1 year, not as a planned repeat test
Kim S.Y	2021	Colonoscopy Versus Fecal Immunochemical Test for Reducing Colorectal Cancer Risk: A Population-Based Case–Control Study	Single sampling over multiple screening rounds
Kwak	2017	Age-adapted Variation in Screening Interval of Fecal Immunochemical Test May Improve its Participation and Colonoscopy Acceptance	Single sampling over multiple screening rounds
Lane	2010	Interval Fecal Immunochemical Testing in a Colonoscopic Surveillance Program Speeds Detection of Colorectal Neoplasia	Single sampling over multiple screening rounds
Lee	2018	Is It Necessary to Repeat Fecal Occult Blood Tests with Borderline Results for Colorectal Cancer Screening?	Repeat samples from the same stool
Levi	2006	A quantitative immunochemical faecal occult blood test is more efficient for detecting significant colorectal neoplasia than a sensitive guaiac test	Data included in further study (Rozen, 2008)
Levi	2007	Can quantification of faecal occult blood predetermine the need for colonoscopy in patients at risk for non-syndromic familial colorectal cancer?	Subgroup analysis of Hazazi, 2010

Levi	2009	Sensitivity, but Not Specifi city, of a Quantitative Immunochemical Fecal Occult Blood Test for Neoplasia Is Slightly Increased by the Use of Low-Dose Aspirin, NSAIDS, and Anticoagulants	Data included in further study (Levi, 2007)
Levi	2010	A quantitative immunochemical fecal occult blood test for colorectal neoplasia	Data included in further study (Rozen, 2010)
Lim	2021	Colorectal cancer in patients with single versus double positive faecal immunochemical test results: A retrospective cohort study from a public tertiary hospital.	No details of FIT used
Liss	2013	Adherence to Repeat Fecal Occult Blood Testing in an Urban Community Health Center Network	Single sampling over multiple screening rounds
Lu	2021	Head-to-head comparison of the test performance of self-administered qualitative vs. laboratory-based quantitative fecal immunochemical tests in detecting colorectal neoplasm.	Single test of multiple FITs
Maclean	2020	Adoption of faecal immunochemical testing for 2-week-wait colorectal patients during the COVID-19 pandemic: an observational cohort study reporting a new service at a regional centre	Single test of two FITs
McNamara	2011	Round one of the Adelaide and Meath Hospital/Trinity College Colorectal Cancer Screening Programme: programme report and analysis based on established international key performance indices	Data included in further study (Kelley, 2013)
Miller	2021	Short-term outcomes of a COVID-adapted triage pathway for colorectal cancer detection	Data included in further study (Gerrard, 2023)
Mosen	2014	Participant uptake of the fecal immunochemical test decreases with the two-sample regimen compared with one-sample FIT.	No data for FIT diagnostic performance
Murphy	2020	Challenges and Approaches to Measuring Repeat Fecal Immunochemical Test for Colorectal Cancer Screening	Single sampling over multiple screening rounds
Nakama	2000	Relationships between a sign of rectal bleeding and the results of an immunochemical occult blood test, and colorectal cancer	No data for FIT diagnostic performance
Nakama	2000	A comparative study of immunochemical fecal tests for detection of colorectal adenomatous polyps.	No data for FIT diagnostic performance of CRC or ACRN at set thresholds
Nakama	2000	Association of diverticulosis coli and vascular ectasias and the results of fecal occult blood test	No data for FIT diagnostic performance of CRC or ACRN at set thresholds
Nakama	2001	Age-Related Cancer Detection Rate and Costs for One Cancer Detected in One Screening by Immunochemical Fecal Occult Blood Test	No data for FIT diagnostic performance
Nishida	2011	Effectiveness of repeated screening using the fecal occult blood test and its impact on reducing false-negative cancer cases	Single sampling over multiple screening rounds
Okada	2020	Colorectal cancer risk factors in asymptomatic Chilean population: a survey of international collaboration between Japan and Chile	Repeat samples from the same stool
Ou	2013	Comparison of the performance of guaiac-based and two immunochemical fecal occult blood tests for identifying advanced colorectal neoplasia in Taiwan	Single test of two FITs
Ozaki	2010	Total Colonoscopy Detects Early Colorectal Cancer More Frequently than Advanced Colorectal Cancer in Patients with Fecal Occult Blood	No details of FIT used

Randel	2021	Colorectal Cancer Screening With Repeated Fecal Immunochemical Test Versus Sigmoidoscopy: Baseline Results From a Randomized Trial	Single sampling over multiple screening rounds
Ribbing Wilén	2018	Fecal immunochemical test in colorectal cancer screening: Colonoscopy findings by different cut-off levels	Data included in further study (Ribbing Wilén, 2019)
Ribe	2022	Impact of time between faecal immunochemical tests in colorectal cancer screening on screening results: A natural experiment	Single sampling over multiple screening rounds
Rozen	2005	Evaluation of a desk top instrument for the automated development and immunochemical quantification of fecal occult blood	Techincal assessment of analyser
Rozen	2009	Identification of colorectal adenomas by a quantitative immunochemical faecal occult blood screening test depends on adenoma characteristics, development threshold used and number of tests performed.	No data for FIT diagnostic performance of CRC or ACRN at set thresholds
Rubeca	2012	Overall evaluation of an immunological latex agglutination system for fecal occult blood testing in the colorectal cancer screening program of Florence	Single test of two FITs
Saito	2020	Efficacy of screening using annual fecal immunochemical test alone versus combined with one-time colonoscopy in reducing colorectal cancer mortality: the Akita Japan population-based colonoscopy screening trial (Akita pop-colon trial)	Single sampling over multiple screening rounds
Santare	2016	Comparison of the yield from two faecal immunochemical tests at identical cut off concentrations - a randomized trial in Latvia.	Single test of two FITs
Symonds	2015	Factors affecting faecal immunochemical test positive rates: demographic, pathological, behavioural and environmental variables	No data for FIT diagnostic performance of CRC or ACRN at set thresholds
Tamin	2020	A 5-year evaluation of using stool-based test for opportunistic colorectal cancer screening in primary health institutions across Malaysia	No details of FIT used
Tao	2013	Well adjusted qualitative immunochemical faecal occult blood tests could be a promising alternative for inexpensive, high-quality colorectal cancer screening	Repeat samples from the same stool
Tepeš	2017	Results of the FIT-based National Colorectal Cancer Screening Program in Slovenia	Data included in further study (Tepeš, 2022)
Toyoshima	2021	Priority stratification for colonoscopy based on two-sample faecal immunochemical test screening: results from a cross-sectional study at an endoscopy clinic in Japan	No data for FIT diagnostic performance
Turvill	2018	Diagnostic accuracy of one or two faecal haemoglobin and calprotectin measurements in patients with suspected colorectal cancer.	No data for FIT diagnostic performance of CRC or ACRN at set thresholds
Van Der Vlugt	2017	Adherence to colorectal cancer screening: four rounds of faecal immunochemical test-based screening	Single sampling over multiple screening rounds
Van Roon	2011	Diagnostic Yield Improves With Collection of 2 Samples in Fecal Immunochemical Test Screening Without Affecting Attendance.	Data included in further study (Schreuders, 2019)
Van Roon	2012	Are Fecal Immunochemical Test Characteristics Influenced by Sample Return Time? A Population-Based Colorectal Cancer Screening Trial	Data included in further study (Schreuders, 2019)
Van Roon	2013	Random comparison of repeated faecal immunochemical testing at different intervals for population-based colorectal cancer screening	Single sampling over multiple screening rounds

Vilkin	2005	Performance Characteristics and Evaluation of an Automated-Developed and Quantitative, Immunochemical, Fecal Occult Blood Screening Test	Data included in further study (Levi, 2007 & Rozen 2010)
Wieten	2018	Equivalent Accuracy of 2 Quantitative Fecal Immunochemical Tests in Detecting Advanced Neoplasia in an Organized Colorectal Cancer Screening Program	Single test of two FITs
Wieten	2020	A Quarter of Participants With Advanced Neoplasia Have Discordant Results From 2-Sample Fecal Immunochemical Tests for Colorectal Cancer Screening.	Single test of two FITs
Woo	2005	A prospective study of a new immunochemical fecal occult blood test in Korean patients referred for colonoscopy	Single test of multiple FITs
Young	2020	Detection of advanced colorectal neoplasia and relative colonoscopy workloads using quantitative faecal immunochemical tests: an observational study exploring the effects of simultaneous adjustment of both sample number and test positivity threshold	Data included in further study (Young, 2020)

Supplementary File S5: Summary of included studies

Author	Population	Number of FIT	Fit Thresholds (µg Hb/g)	Time Between FIT Samples	Quantitative / Qualitative	Analyser Brand	Number Sent FIT	Number of FIT Complete	FIT Participation Rate	Intended Investigation Criteria	Investigation Methods	Number Investigated	CRC Diagnosed	ACRN Diagnosed	Prevalence of CRC in Population Completing FIT	Prevalence of ACRN in Population Completing FIT	Prevalence of CRC in Population Investigated	Prevalence of ACRN in Population Investigated	Data Utilised For Diagnostic Performance
Abdullah, 2020	Asymptomatic, Age 35-65	2	10	Within 5 Days	Qualitative	OC Light	2264	1803	79.6%	At Least One Positive FIT	Colonoscopy	125	6	n/a	0.3%	n/a	4.8%	n/a	No
Cai, 2016	Asymptomatic, Age 40-74	2	50	Within 1 Week	Qualitative	Hemosure	31963	24419	76.4%	At Least One Positive FIT	Colonoscopy	1430	39	138	0.2%	0.6%	2.7%	9.7%	No
Chubak, 2013	Asymptomatic, Age 50-74	2	50	Consecutive Days	Qualitative	InSure	750	445	59.3%	At Least One Positive FIT	Colonoscopy	21	0	n/a	0.0%	n/a	0.0%	n/a	No
Cole, 2003	Asymptomatic, Age 50-69	2 (InSure)<br>3 (FlexSure)	InSure: 50<br>FlexSure: 300	Consecutive Bowel Motions	Qualitative	InSure<br>FlexSure OBT	InSure: 606<br>FlexSure: 606	InSure: 240<br>FlexSure: 185	Insure: 39.6%<br>FlexSure: 30.5%	At Least One Positive FIT	Colonoscopy	35	n/a	n/a	n/a	n/a	n/a	n/a	No
Dancourt, 2008	Asymptomatic, Age 50-74	2	50	Consecutive Bowel Motions	Qualitative	Instant-view	n/a	17215	n/a	At Least One Positive FIT or gFOBT	Colonoscopy	1205	57	332	0.3%	1.9%	4.7%	27.6%	No
Faivre, 2012	Asymptomatic, Age 50-74	2	30, 40, 50, 60	Consecutive Days	Quantitative	OC Sensor<br>& FOB-Gold	n/a	OC Sensor: 33690<br>FOB-Gold: 32215	n/a	At Least One Positive FIT or gFOBT	Colonoscopy	OC Sensor: 1181<br>FOB-Gold: 1527	OC Sensor: 92<br>FOB-Gold: 91	OC Sensor: 495<br>FOB-Gold: 407	OC Sensor: 0.3%<br>FOB-Gold: 0.3%	OC Sensor: 1.5%<br>FOB-Gold: 1.3%	OC Sensor: 7.8%<br>FOB-Gold: 6.0%	OC Sensor: 41.9%<br>FOB-Gold: 26.7%	No
Fu, 2009	Asymptomatic, Age 40 and Over	2	20	Consecutive Days	Quantitative	OC Sensor	751	547	72.8%	At Least One Positive FIT	Colonoscopy	52	3	15	0.5%	2.7%	5.8%	28.8%	No
Grazzini, 2009	Asymptomatic, Age 50-69	2	16, 20, 24	Consecutive Bowel Motions	Quantitative	OC Sensor Micro	36646	20596	56.2%	At Least One Positive FIT	Colonoscopy	1465	69	534	0.3%	2.6%	4.7%	36.5%	No
Guittet, 2009	Asymptomatic, Age 50-74	2	67	Consecutive Days	Quantitative	Magstream 1000	n/a	20322	n/a	At Least One Positive FIT or gFOBT	Colonoscopy	1277	41	291	0.2%	1.4%	3.2%	22.8%	No
Hernandez, 2014	Asymptomatic, Age 50-69	2	10, 15, 20, 23, 30, 40	Consecutive Days	Quantitative	OC Sensor	n/a	833	n/a	All Patients	Colonoscopy	779	5	97	0.6%	11.4%	0.6%	12.5%	Yes
Kapidzic, 2017	Asymptomatic, Age 50-74	2	10	Consecutive Days	Quantitative	OC Sensor Micro	2636	1582	60.0%	At Least One Positive FIT	Colonoscopy	128	4	27	0.3%	1.7%	3.1%	21.1%	No
Kelley, 2013	Asymptomatic, Age 50-75	2	20	Between 2-6 Days	Quantitative	OC Sensor	9704	5023	51.8%	At Least One Positive FIT	Colonoscopy	419	17	149	0.3%	3.0%	4.1%	35.6%	No
Launoy, 2005	Asymptomatic, Age 50-74	2	67	Consecutive Days	Quantitative	Magstream 1000	n/a	7421	n/a	At Least One Positive FIT, Negative FIT Observed for Minimum 12 Months, 80% 2 Years	Colonoscopy or Registry Follow-up	7421	28	n/a	0.4%	n/a	0.4%	n/a	Yes
Levi, 2011	Asymptomatic, Age 50-75	3	14	Consecutive Days	Quantitative	OC Sensor Micro	1536	1224	79.7%	At Least One Positive FIT, 2 Year Follow-up CRC Data	Colonoscopy or Registry Follow-up	1224[*]	6	n/a	0.5%	n/a	0.5%	n/a	Yes
Liles, 2018	Asymptomatic, Age 49-75	2	10, 15, 20, 25, 30	Within 1 Week	Quantitative	OC Auto	4718	3854	81.7%	All Patients	Colonoscopy	2771	2	211	0.1%	5.5%	0.1%	7.6%	Yes
Moosavi, 2016	Asymptomatic, Age 50-75	2	10, 15, 20, 25, 31	Consecutive Bowel Motions	Quantitative	OC Auto	n/a	17031	n/a	At Least One Positive FIT	Colonoscopy	1403	58	477	0.3%	2.8%	4.1%	34.0%	No
Nakama (A), 2000	Asymptomatic, Age 40 and Over	3	10, 15, 20, 25, 32	Consecutive Days	Qualitative	Monohaem	n/a	3300	n/a	All Patients	Colonoscopy	3300	17	n/a	0.5%	n/a	0.5%	n/a	No
Nakama (B), 2000	Asymptomatic, Age 40-60	2	10, 15, 20, 25, 33	Consecutive Days	Qualitative	Iatro Hemcheck	n/a	17664	n/a	All Patients	Colonoscopy	17664	96	n/a	0.5%	n/a	0.5%	n/a	No
Nakama, 2002	Asymptomatic	2	10, 15, 20, 25, 34	Consecutive Days	Qualitative	OC Hemodia	n/a	44821	n/a	At Least One Positive FIT	Colonoscopy	2330	74	n/a	0.2%	n/a	3.2%	n/a	No
Nakazato, 2006	Asymptomatic	2	10, 15, 20, 25, 35	Consecutive Days	Qualitative	OC Hemodia	n/a	3090[§]	n/a	All Patients	Colonoscopy	3090	19	72	0.6%	2.3%	0.6%	2.3%	No
Okada, 2020	Asymptomatic, Age 50-75	2	10, 15, 20, 25, 36	Consecutive Days	Quantitative	OC Hemodia	26444	24285	91.8%	At Least One Positive FIT, or FH CRC	Colonoscopy	4196	20	n/a	0.8%	n/a	4.8%	n/a	No
Park, 2010	Asymptomatic, Age 50-74	3	10, 15, 20, 25, 37	Consecutive Bowel Motions	Quantitative	OC Sensor Micro	891	836	93.8%	All Patients	Colonoscopy	770	13	72	1.6%	8.6%	1.7%	9.4%	Yes
Raginel, 2013	Asymptomatic, Age 50-74	2	10, 15, 20, 25, 38	Consecutive Bowel Motions	Quantitative	OC Sensor & Magstream 1000	19797	18563	93.8%	At Least One Positive FIT or gFOBT	Colonoscopy	1075	47	287	0.3%	1.5%	4.4%	26.7%	No
Ribbing Wilén, 2019	Asymptomatic, Age 60	2	10, 15, 20, 25, 39	Consecutive Bowel Motions	Quantitative	OC Sensor	1032	840	81.4%	All Patients	Colonoscopy	806	n/a	81	n/a	9.6%	n/a	10.0%	Yes
Rutka, 2020	Asymptomatic, Age 50-70	2	10, 15, 20, 25, 40	Consecutive Days	Quantitative	OC Sensor	22130	10273	46.4%	At Least One Positive FIT	Colonoscopy	766	42	n/a	0.4%	n/a	5.5%	n/a	No
Sakata, 2014	Asymptomatic, Age 40 and Over	2	10, 15, 20, 25, 41	Consecutive Days	Quantitative	OC Sensor	n/a	55595	n/a	At Least One Positive FIT	Colonoscopy	4615	114	n/a	0.2%	n/a	2.5%	n/a	No
Schreuders, 2019	Asymptomatic, Age 50-74	2	10, 15, 20, 25, 42	Consecutive Days	Quantitative	OC Sensor Micro	Round 1: 3057<br>Round 2: 2579	1875<br>1582	61.3%<br>61.3%	At Least One Positive FIT	Colonoscopy	Round 1: 226<br>Round 2: 128	Round 1: 13<br>Round 2: 4	Round 1: 77<br>Round 2: 27	0.7%<br>0.3%	4.1%<br>1.7%	5.8%<br>3.1%	34.1%<br>21.1%	No
							Round 3: 2287	1474	64.5%			Round 3: 137	Round 3: 6	Round 3: 20	0.4%	1.4%	4.4%	14.6%	
							Round 4: 1845	1171	63.5%			Round 4: 116	Round 4: 3	Round 4: 23	0.3%	2.0%	2.6%	19.8%	
Sekiguchi, 2021	Asymptomatic, Age 49-79	2	10, 20, 30, 40	Consecutive Days	Quantitative	OC Sensor	1367	1345	98.4%	All Patients	Colonoscopy	1191	10	112	0.7%	8.3%	0.8%	9.4%	Yes
Shapiro, 2017	Asymptomatic, Age 50-74	2	50	Consecutive Days	Qualitative	InSure	n/a	987	n/a	All Patients	Colonoscopy	987	n/a	53	n/a	5.4%	n/a	5.4%	No
Shuhaibar, 2011	Asymptomatic, Age 50 and Over	2	20	Consecutive Days	Quantitative	OC Sensor	436	254	58.3%	At Least One Positive FIT	Colonoscopy	16	4	n/a	1.6%	n/a	25.0%	n/a	No
Smith, 2006	Asymptomatic	2	50	Consecutive Bowel Motions	Qualitative	InSure	n/a	2351	n/a	At Least One Positive FIT or Clinician Deems High-Risk	Colonoscopy	431	17	45	0.7%	1.9%	3.9%	10.4%	No
Tan, 2013	Asymptomatic, Age 50 and Over	2	10	Within 1 Week	Qualitative	OC Light	20989	8156	38.9%	At Least One Positive FIT	Colonoscopy	494	33	n/a	0.4%	n/a	6.7%	n/a	No
Telford, 2016	Asymptomatic, Age 50-74	2	20	Consecutive Bowel Motions	Quantitative	OC Auto	Round 1: 19864<br>Round 2: 6255	Round 1: 16234<br>Round 2: 5378	Round 1: 81.7%<br>Round 2: 86.0%	At Least One Positive FIT	Colonoscopy	1555	76	619	0.4%	2.9%	4.9%	39.8%	No<br>No
Tepeš, 2014	Asymptomatic, Age 64-68	2	67	Consecutive Bowel Motions	Quantitative	Magstream 1000	3117	2829	90.8%	At Least One Positive FIT	Colonoscopy	217	15	75	0.5%	2.7%	6.9%	34.6%	No
Tepeš, 2022	Asymptomatic, Age 50-74	2	67	Consecutive Bowel Motions	Quantitative	Magstream 1000<br>& OC Sensor	n/a	Round 1: 303343<br>Round 2: 289070	n/a	At Least One Positive FIT	Colonoscopy	Round 1: 862<br>Round 2: 469	n/a	n/a	Round 1: 0.3%<br>Round 2: 0.2%	n/a	Round 1: 6.0%<br>Round 2: 3.3%	n/a	No
								Round 3: 298951				Round 3: 405			Round 3: 0.1%		Round 3: 2.6%		
Toume-Garcia, 2022	Asymptomatic, Age 50-69	2	20	Consecutive Bowel Motions	Quantitative	HM-JACKarc	n/a	114049	n/a	At Least One Positive FIT	Colonoscopy	8871	266	n/a	0.2%	n/a	3.0%	n/a	No
Wang, 2022	Asymptomatic, Age 40-74	2	1-5	One Week Apart	Qualitative	ABON FOB	19131	14437	75.5%	At Least One Positive FIT	Colonoscopy	1091	10	115	0.1%	0.8%	0.9%	10.5%	No
Wong M.C.S, 2015	Asymptomatic, Age 50-74	2	50	Consecutive Bowel Motions	Qualitative	Hemosure	n/a	5343	n/a	All Patients	Colonoscopy	5343	14	165	0.3%	3.1%	0.3%	3.1%	No
Yang, 2011	Asymptomatic	2	20	Consecutive Days	Quantitative	OC Sensor Micro	n/a	5919	n/a	At Least One Positive FIT	Colonoscopy	241	16	n/a	0.3%	n/a	6.6%	n/a	No
Ye, 2017	Asymptomatic, Age 40-74	2	50	Within 1 Week	Qualitative	Hemosure	142124	120832	85.0%	At Least One Positive FIT or Cancer Registry Follow-up	Colonoscopy or Registry Follow-up	119377	291	n/a	0.2%	n/a	0.2%	n/a	No
Auge, 2013	Surveillance or Symptomatic	2	8, 16, 24	Consecutive Bowel Motions	Quantitative	Kroma iT	n/a	210	n/a	All Patients	Colonoscopy	210	n/a	25	n/a	11.9%	n/a	11.9%	Yes
Auge, 2016	Surveillance or Symptomatic	2	>0, 10, 20, 30 ,40	Consecutive Bowel Motions	Quantitative	HM-JACKarc	n/a	208	n/a	All Patients	Colonoscopy	208	2	29	1.0%	13.9%	1.0%	13.9%	Yes
Auge, 2018	Surveillance or Symptomatic	2	10, 20, 30, 40, 50, 60	Consecutive Bowel Motions	Quantitative	SENTiFIT	n/a	487	n/a	All Patients	Colonoscopy	487	12	71	2.5%	14.6%	2.5%	14.6%	Yes
Castro, 2013	High Risk Screening of FDR with CRC	2	10, 15, 20, 23, 30, 40	Within 1 Week	Quantitative	OC Sensor	635	619	97.5%	All Patients	Colonoscopy	595	6	64	1.0%	10.3%	1.0%	10.8%	Yes
Chew, 2009	Screening, Surveillance or Symptomatic	2	20	Consecutive Days	Quantitative	OC Sensor Micro	1048	768	73.3%	At Least One Positive FIT	Colonoscopy	47	1	n/a	0.1%	n/a	2.1%	n/a	No
Cruz-Correa, 2007	Screening, Surveillance, Previous CRC or Significant Family History	3	2.0mg Hb/100ml	Within 1 Week	Qualitative	EZ Detect	207	207	100.0%	All Patients	Colonoscopy	207	0	7	0.0%	3.4%	0.0%	3.4%	No
Guimarães, 2019	Screening, Surveillance or Symptomatic	3	4ng/ml	Consecutive Days	Qualitative	ColonView	774	581	75.1%	All Patients	Colonoscopy	368	37	85	6.4%	14.6%	10.1%	23.1%	No
Hazazi, 2010	High Risk Screening or Surveillance	3	10, 15, 20	Consecutive Bowel Motions	Quantitative	OC Sensor Micro	1376	1071	77.8%	All Patients	Colonoscopy	1000	8	72	0.7%	6.7%	0.8%	7.2%	Yes
Kovarova, 2012	Screening, Surveillance or Symptomatic	2	10, 15, 20, 25, 30	Consecutive Bowel Motions	Quantitative	OC Sensor	815	709	87.0%	All Patients	Colonoscopy	669[¤]	35	63	4.9%	8.9%	5.2%	9.4%	Yes
Li, 2006	Screening, Surveillance or Symptomatic	3	50	Consecutive Bowel Motions	Qualitative	Hemosure	n/a	323	n/a	All Patients	Colonoscopy	323	49	83	15.2%	25.7%	15.2%	25.7%	No
Oort, 2011	Screening, Surveillance or Symptomatic	2	10, 15, 20, 30, 40	Consecutive Days	Quantitative	OC Sensor Micro	n/a	1589	n/a	All Patients	Colonoscopy	1081[**]	50	139	3.1%	8.7%	4.5%	12.4%	Yes
Randell, 2013	High Risk Screening or Symptomatic	2	10, 20	Consecutive Days	Quantitative	Hemo Techt NS Plus	354	249	70.3%	All Patients	Colonoscopy	249	2	6	0.8%	2.4%	0.8%	2.4%	Yes
Redwood, 2014	Asymptomatic or Surveillance	2	50	Consecutive Bowel Motions	Qualitative	InSure	397	368	92.7%	All Patients	Colonoscopy	304	4	28	1.1%	7.6%	1.3%	9.2%	No
Rozen, 2009	High Risk Screening, Surveillance or Symptomatic	3	10, 15, 20, 25, 30, 40	Consecutive Days	Quantitative	OC Sensor Micro	n/a	330	n/a	All Patients	Colonoscopy	330	6	32	1.8%	9.7%	1.8%	9.7%	Yes
Rozen, 2010	High Risk Screening, Surveillance or Symptomatic	3	10, 15, 20, 25, 30, 40	Consecutive Days	Quantitative	OC Sensor Micro	2352	1682	71.5%	All Patients	Colonoscopy	1682	20	149	1.2%	8.9%	1.2%	8.9%	Yes
Terhaar sive Droste, 2012	High Risk Screening or Surveillance	2	10	Consecutive Days	Quantitative	OC Sensor	1302	1299	99.8%	All Patients	Colonoscopy	1T: 1041 / 2T: 451[+]	5	106	0.4%	8.2%	0.5%	10.2%	Yes
Vasilyev, 2015	Screening, Surveillance or Symptomatic	3	4ng/ml	Consecutive Days	Qualitative	ColonView	n/a	300	n/a	All Patients	Colonoscopy	300	95	n/a	31.7%	n/a	31.7%	n/a	No
Wong B.C, 2003	Surveillance or Symptomatic	3	300	Consecutive Bowel Motions	Qualitative	FlexSure OBT	136	135	99.3%	All Patients	Colonoscopy	135	9	11	6.7%	8.1%	6.7%	8.1%	No
Wong W.M, 2003	Surveillance or Symptomatic	2	67	Consecutive Bowel Motions	Quantitative	Magstream 1000	n/a	250	n/a	All Patients	Colonoscopy	250	7	26	2.8%	10.4%	2.8%	10.4%	Yes
Wu, 2014	Screening, Surveillance or Symptomatic	3	6	Within 1 Week	Qualitative	Acon Biotech	n/a	513	n/a	All Patients	Colonoscopy	513	52	122	10.1%	23.8%	10.1%	23.8%	No
Young, 2020	High Risk Screening or Surveillance	2	10, 20, 25, 40, 80	Consecutive Bowel Motions	Quantitative	OC Sensor	19229	12710	66.1%	All Patients	Colonoscopy	4037	21	626	0.2%	4.9%	0.5%	15.5%	Yes
	**Symptomatic**																		
Fernández-Bañares, 2019	Symptomatic	3	11	Within 1 Week	Quantitative	iFOB	897	867	96.7%	All Patients	Colonoscopy	867	67	171	7.7%	19.7%	7.7%	19.7%	Yes
Gerrard, 2023	Symptomatic	2	10, 20[▪]	Median 13 Days	Quantitative	HM-JACKarc	4354	3890	89.3%	All Patients	Colonoscopy or CTC	2637	88	185	2.3%	4.8%	3.3%	7.0%	Yes
Högberg, 2020	Symptomatic	3	Actim Fecal Blood: 25-50<br>Analyz FOB: 2	Consecutive Days	Qualitative	Actim Fecal Blood<br>Analyz FOB	n/a	Actim Fecal Blood: 4232<br>Analyz FOB: 7889	n/a	At Least One Positive FIT or Cancer Registry Follow-up	Colonoscopy or Registry Follow-up	Actim Fecal Blood: 4232<br>Analyz FOB: 7889	Actim Fecal Blood: 79<br>Analyz FOB: 158	n/a<br>n/a	Actim Fecal Blood: 1.9%<br>Analyz FOB: 2.0%	n/a<br>n/a	Actim Fecal Blood: 1.9%<br>Analyz FOB: 2.0%	n/a<br>n/a	No
			Chemtrue FOB: 40ng/ml			Chemtrue FOB		Chemtrue FOB: 1756				Chemtrue FOB: 1756	Chemtrue FOB: 29	n/a	Chemtrue FOB: 1.7%	n/a	Chemtrue FOB: 1.7%	n/a	
			Diaquick FOB: 5			Diaquick FOB		Diaquick FOB: 1751				Diaquick FOB: 1751	Diaquick FOB: 38	n/a	Diaquick FOB: 2.2%	n/a	Diaquick FOB: 2.2%	n/a	
								Diaquick FOB or Analyz FOB: 161				Diaquick FOB or Analyz FOB: 161	Diaquick FOB or Analyz FOB: 0	n/a	Diaquick FOB or Analyz FOB: 0.0%	n/a	Diaquick FOB or Analyz FOB: 0.0%	n/a	
Hunt N., 2022	Symptomatic	2	10	Different Bowel Motions	Quantitative	OC Sensor	n/a	28622	n/a	All Patients Cancer Registry Follow-up	Registry Follow-up	28622	319	n/a	1.1%	n/a	1.1%	n/a	Yes
Mattar, 2020	Symptomatic	2	10	Consecutive Days	Quantitative	OC Sensor	474	333	70.3%	All Patients	Colonoscopy	1T: 172 / 2T: 117	1T: 6 / 2T: 8	1T: 32 / 2T: 20	4.2%	15.6%	1T: 3.5% / 2T: 6.8%	1T: 18.0% / 2T: 17.1%	Yes
Oono, 2010	Symptomatic	2	19	Within 5 Days	Quantitative	Hemo Techt NS Plus	n/a	1200	n/a	All Patients	Colonoscopy	1073	91	n/a	n/a	n/a	8.5%	n/a	Yes
Smith , 2006	Symptomatic	2	50	Consecutive Bowel Motions	Qualitative	InSure	n/a	161	n/a	All Patients	Colonoscopy	161	7	23	4.3%	14.3%	4.3%	14.3%	No
Tsapoumas, 2020	Symptomatic	2	10,15,20	Consecutive Days	Quantitative	QuikRead	355	277	78.0%	All Patients	Colonoscopy	242[§]	13	28	4.7%	10.1%	5.4%	11.6%	Yes

Supplementary File S6: (A) FIT positivity by number of tests and thresholds utilised. (B) Two test positivity at 10µg by FIT analyser

(A)

Positivity Threshold	Population	Author	FIT (µg Hb/g)	1T	2T	3T
**FIT <10µg Hb/g**	Mixed	Auge, 2016	>0	90.4%	97.1%	
	Symptomatic	Högberg, 2020	2			28.3%
	Asymptomatic	Wang, 2022	1-5		14.2%	
	Symptomatic	Högberg, 2020	5			21.9%
	Mixed	Wu, 2014	6		50.7%	
	Mixed	Auge, 2013	8	14.4%	20.5%	
**FIT 10µg Hb/g**	Asymptomatic	Abdullah, 2020	10		13.1%	
	Asymptomatic	Hernandez, 2014	10	8.6%	13.0%	
	Asymptomatic	Kapidzic, 2017	10		8.3%	
	Asymptomatic	Liles, 2018	10	7.6%	12.0%	
	Asymptomatic	Ribbing Wilén, 2019	10	8.6%	12.7%	
	Asymptomatic	Schreuders, 2019 (Round 1)	10		12.7%	
	Asymptomatic	Schreuders, 2019 (Round 2)	10		8.3%	
	Asymptomatic	Schreuders, 2019 (Round 3)	10		9.7%	
	Asymptomatic	Schreuders, 2019 (Round 4)	10		10.8%	
	Asymptomatic	Tan, 2013	10		8.1%	
	Asymptomatic	Sekiguchi, 2021	10	6.8%	10.6%	
	Mixed	Auge, 2016	10	15.8%	23.4%	
	Mixed	Auge, 2018	10	19.7%	25.9%	
	Mixed	Castro, 2013	10	8.9%	11.9%	
	Mixed	Hazazi, 2010	10	9.3%	12.8%	16.7%
	Mixed	Kovarova, 2012	10	18.7%	24.0%	
	Mixed	Oort, 2011	10	15.9%	21.3%	
	Mixed	Randell, 2013	10	25.3%	34.5%	
	Mixed	Rozen, 2009	10	10.6%	14.0%	19.1%
	Mixed	Rozen, 2010	10	9.7%	13.8%	16.5%
	Mixed	Terhaar sive Droste, 2012	10	11.0%	19.3%	
	Mixed	Young, 2020	10	7.9%	12.1%	
	Symptomatic	Gerrard, 2023	10	23.6%	31.1%	
	Symptomatic	Hunt N., 2022	10		33.8%	
	Symptomatic	Mattar, 2020	10		19.7%	
	Symptomatic	Tsapournas, 2020	10		31.8%	

**FIT 11-14µg Hb/g**	Symptomatic	Fernández-Bañares, 2019	11		37.6%	
	Asymptomatic	Levi, 2011	14		12.7%	
**FIT 15µg Hb/g**	Asymptomatic	Hernandez, 2014	15	7.8%	11.4%	
	Asymptomatic	Liles, 2018	15	5.1%	8.1%	
	Asymptomatic	Park, 2010	15	7.9%	10.0%	12.3%
	Mixed	Castro, 2013	15	7.4%	10.3%	
	Mixed	Hazazi, 2010	15	7.2%	9.6%	12.2%
	Mixed	Kovarova, 2012	15	15.8%	20.7%	
	Mixed	Oort, 2011	15	13.7%	17.2%	
	Mixed	Rozen, 2009	15	8.8%	10.9%	15.2%
	Mixed	Rozen, 2010	15	7.6%	10.6%	13.1%
	Symptomatic	Tsapournas, 2020	15	22.3%	26.4%	
**FIT 16-19µg Hb/g**	Asymptomatic	Grazzini, 2009	16	5.5%	8.0%	
	Asymptomatic	Nakazato, 2006	16		13.1%	
	Mixed	Auge, 2013	16	12.3%	17.1%	
	Symptomatic	Oono, 2010	19		18.8%	
**FIT 20µg Hb/g**	Asymptomatic	Fu, 2009	20		10.4%	
	Asymptomatic	Grazzini, 2009	20	4.5%	6.7%	
	Asymptomatic	Hernandez, 2014	20	7.1%	10.5%	
	Asymptomatic	Kelley, 2013	20	6.9%	10.2%	
	Asymptomatic	Liles, 2018	20	4.2%	6.5%	
	Asymptomatic	Moosavi, 2016	20		9.3%	
	Asymptomatic	Nakama (A), 2000	20	3.8%	5.1%	5.8%
	Asymptomatic	Okada, 2020	20		15.1%	
	Asymptomatic	Park, 2010	20	7.4%	9.1%	11.4%
	Asymptomatic	Rutka, 2020	20		13.1%	
	Asymptomatic	Sakata, 2014	20		10.5%	
	Asymptomatic	Sekiguchi, 2021	20	5.3%	8.0%	
	Asymptomatic	Shuhaibar, 2011	20		6.3%	
	Asymptomatic	Telford, 2016 (Round 1)	20		8.6%	
	Asymptomatic	Telford, 2016 (Round 2)	20		6.8%	
	Asymptomatic	Tourne-Garcia, 2022	20		8.3%	
	Asymptomatic	Yang, 2011	20		5.3%	
	Mixed	Auge, 2016	20	10.5%	17.2%	
	Mixed	Auge, 2018	20	15.7%	21.0%	
	Mixed	Castro, 2013	20	6.2%	8.6%	
	Mixed	Chew, 2009	20		6.4%	
	Mixed	Hazazi, 2010	20	5.6%	8.0%	10.5%

	Mixed	Kovarova, 2012	20	14.9%	18.7%	
	Mixed	Oort, 2011	20	11.8%	15.0%	
	Mixed	Randell, 2013	20	16.9%	21.3%	
	Mixed	Rozen, 2009	20	7.0%	9.1%	12.7%
	Mixed	Rozen, 2010	20	6.1%	8.7%	10.9%
	Mixed	Young, 2020	20	4.4%	7.0%	
	Symptomatic	Gerrard, 2023	20	19.1%	25.0%	
	Symptomatic	Tsapournas, 2020	20	17.4%	21.9%	
**FIT 21-29µg Hb/g**	Asymptomatic	Hernandez, 2014	23	6.5%	10.1%	
	Mixed	Castro, 2013	23	5.7%	7.7%	
	Asymptomatic	Grazzini, 2009	24	4.0%	5.9%	
	Mixed	Auge, 2013	24	11.4%	15.8%	
	Asymptomatic	Liles, 2018	25	3.6%	5.4%	
	Mixed	Kovarova, 2012	25	12.7%	16.7%	
	Mixed	Rozen, 2009	25	6.0%	7.6%	10.0%
	Mixed	Rozen, 2010	25	5.5%	7.7%	9.5%
	Mixed	Young, 2020	25	3.7%	5.9%	
**FIT 30µg Hb/g**	Asymptomatic	Faivre, 2012 (OC Sensor)	30	2.5%	3.7%	
	Asymptomatic	Faivre, 2012 (FOB-Gold)	30	3.3%	5.2%	
	Asymptomatic	Hernandez, 2014	30	6.2%	9.0%	
	Asymptomatic	Liles, 2018	30	3.0%	4.6%	
	Asymptomatic	Raginel, 2013	30		4.0%	
	Asymptomatic	Sekiguchi, 2021	30	4.3%	6.3%	
	Mixed	Auge, 2016	30	10.0%	16.7%	
	Mixed	Auge, 2018	30	13.5%	16.8%	
	Mixed	Castro, 2013	30	5.2%	7.1%	
	Mixed	Kovarova, 2012	30	12.2%	15.8%	
	Mixed	Oort, 2011	30	9.7%	12.4%	
	Mixed	Rozen, 2009	30	6.0%	7.6%	9.7%
	Mixed	Rozen, 2010	30	4.8%	7.1%	9.0%
**FIT 40µg Hb/g**	Asymptomatic	Faivre, 2012 (OC Sensor)	40	2.0%	3.0%	
	Asymptomatic	Faivre, 2012 (FOB-Gold)	40	2.8%	4.4%	
	Asymptomatic	Hernandez, 2014	40	5.8%	7.6%	
	Asymptomatic	Nakama, 2002	40		5.2%	
	Asymptomatic	Sekiguchi, 2021	40	3.9%	5.3%	
	Mixed	Auge, 2016	40	9.1%	14.8%	
	Mixed	Auge, 2018	40	12.4%	15.6%	
	Mixed	Castro, 2013	40	4.9%	6.6%	

	Mixed	Oort, 2011	40	8.7%	10.8%	
	Mixed	Rozen, 2009	40	5.7%	7.0%	8.8%
	Mixed	Rozen, 2010	40	4.4%	6.4%	8.0%
	Mixed	Young, 2020	40	2.4%	4.0%	
**FIT ≧50μg Hb/g**	Asymptomatic	Cai, 2016	50	4.7%	7.2%	
	Asymptomatic	Chubak, 2013	50		4.7%	
	Asymptomatic	Cole, 2003	50		6.8%	
	Asymptomatic	Dancourt, 2008	50		6.9%	
	Asymptomatic	Faivre, 2012 (OC Sensor)	50	1.7%	2.6%	
	Asymptomatic	Faivre, 2012 (FOB-Gold)	50	2.4%	3.8%	
	Asymptomatic	Shapiro, 2017	50		4.5%	
	Asymptomatic	Smith, 2006	50		5.6%	
	Asymptomatic	Wong M.C.S, 2015	50	7.1%	8.9%	
	Asymptomatic	Ye, 2017	50		9.8%	
	Mixed	Auge, 2018	50	11.2%	14.3%	
	Mixed	Li, 2006	50		16.4%	23.8%
	Mixed	Redwood, 2014	50		10.5%	
	Symptomatic	Högberg, 2020	25-50			30.9%
	Symptomatic	Smith, 2006	50		23.0%	
	Asymptomatic	Faivre, 2012 (OC Sensor)	60	1.5%	2.3%	
	Asymptomatic	Faivre, 2012 (FOB-Gold)	60	2.1%	3.3%	
	Mixed	Auge, 2018	60	11.0%	13.7%	
	Asymptomatic	Guittet, 2009	67	4.2%	6.4%	
	Asymptomatic	Launoy, 2005	67		5.8%	
	Asymptomatic	Tepeš, 2014	67		7.5%	
	Asymptomatic	Tepeš, 2022 (Round 1)	67		6.1%	
	Asymptomatic	Tepeš, 2022 (Round 2)	67		6.0%	
	Asymptomatic	Tepeš, 2022 (Round 3)	67		6.0%	
	Mixed	Wong W.M, 2003	67		20.4%	
	Mixed	Young, 2020	80	1.4%	2.3%	
	Asymptomatic	Cole, 2003	300		4.6%	
	Asymptomatic	Raginel, 2013	180		3.8%	
	Mixed	Wong B.C, 2003	300		14.1%	

(B)

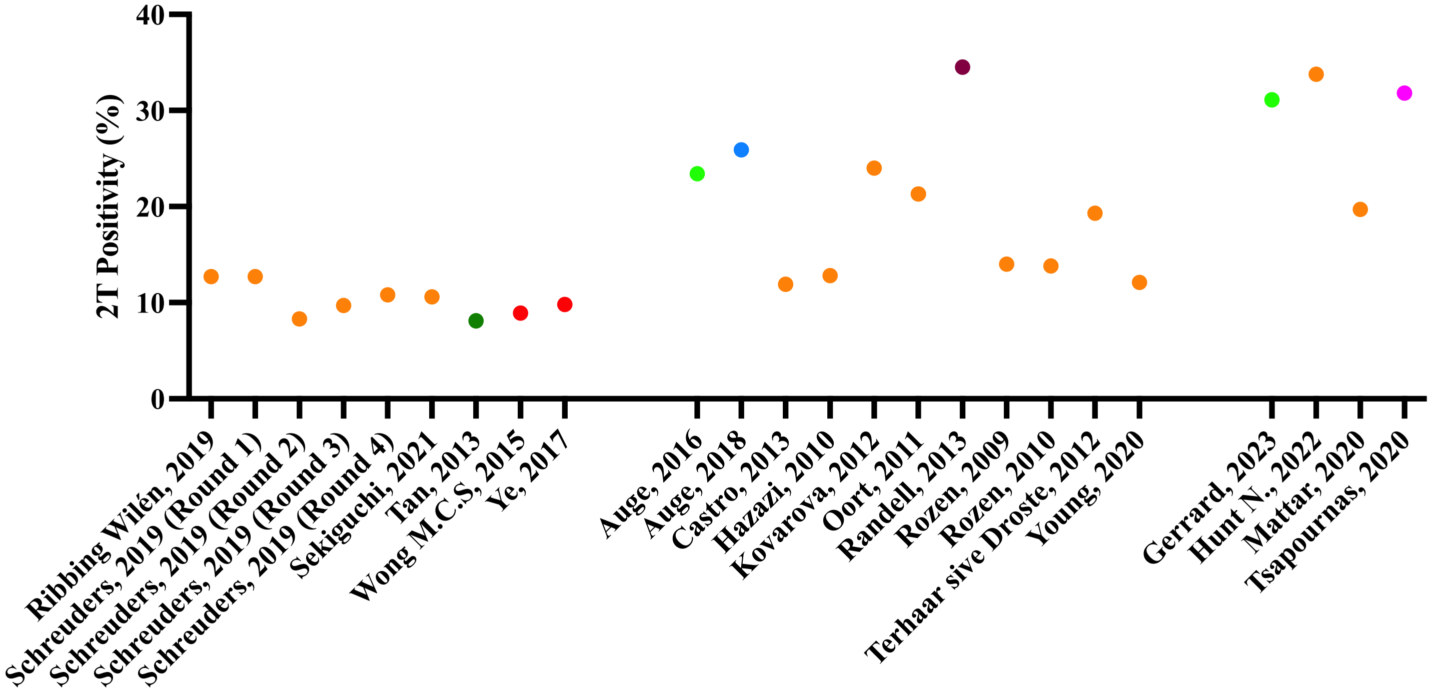
2T Positivity (%)
40
30
20
10
0
Ribbing Wilén, 2019
Schreuders, 2019 (Round 1)
Schreuders, 2019 (Round 2)
Schreuders, 2019 (Round 3)
Schreuders, 2019 (Round 4)
Sekiguchi, 2021
Tan, 2013
Wong M.C.S, 2015
Ye, 2017
Auge, 2016
Auge, 2018
Castro, 2013
Hazazi, 2010
Kovarova, 2012
Oort, 2011
Randell, 2013
Rozen, 2009
Rozen, 2010
Terhaar sive Droste, 2012
Young, 2020
Gerrard, 2023
Hunt N., 2022
Mattar, 2020
Tsapournas, 2020

Supplementary File S7: False positive rates at differing thresholds and populations for (A) CRC and (B) ACRN

(A) CRC

10µg Hb/g - Mixed

Auge, 2018: FPR = 0.179, CI = [0.143, 0.215]
Castro, 2014: FPR = 0.110, CI = [0.084, 0.136]
Hazazi, 2010: FPR = 0.118, CI = [0.099, 0.137]
Kovarova, 2012: FPR = 0.185, CI = [0.148, 0.222]
Randell, 2013: FPR = 0.340, CI = [0.281, 0.399]
Rozen, 2010: FPR = 0.128, CI = [0.111, 0.145]
Terhaar sive Droste, 2012: FPR = 0.192, CI = [0.154, 0.230]

10µg Hb/g - Symptomatic

Gerrard, 2023: FPR = 0.288, CI = [0.270, 0.306]
Hunt, 2022: FPR = 0.331, CI = [0.323, 0.339]
Mattar, 2020: FPR = 0.156, CI = [0.089, 0.223]
Tsapournas, 2020: FPR = 0.286, CI = [0.227, 0.345]

15µg Hb/g - Mixed

Castro, 2014: FPR = 0.093, CI = [0.071,0.119]
Hazazi, 2010: FPR = 0.086, CI = [0.065,0.112]
Kovarova, 2012: FPR = 0.152, CI = [0.122,0.188]
Rozen, 2010: FPR = 0.096, CI = [0.074,0.111]

20µg Hb/g - Mixed

Auge, 2018: FPR = 0.140, CI = [0.112,0.170]
Castro, 2014: FPR = 0.076, CI = [0.038,0.103]
Hazazi, 2010: FPR = 0.070, CI = [0.043,0.090]
Kovarova, 2012: FPR = 0.130, CI = [0.064,0.179]
Randell, 2013: FPR = 0.206, CI = [0.130,0.263]
Rozen, 2010: FPR = 0.079, CI = [0.057,0.094]

(B) ACRN

10µg Hb/g - Asymptomatic

Hernandez, 2014: FPR = 0.088, CI = [0.066, 0.110]
Liles, 2018: FPR = 0.105, CI = [0.093, 0.117]
Masau, 2021: FPR = 0.082, CI = [0.066, 0.098]
Ribbing Wilén, 2019: FPR = 0.112, CI = [0.090, 0.134]

10µg Hb/g - Mixed

Auge, 2018: FPR = 0.214, CI = [0.175, 0.253]
Castro, 2013: FPR = 0.073, CI = [0.052, 0.094]
Hazazi, 2010: FPR = 0.090, CI = [0.073, 0.107]
Kovarova, 2012: FPR = 0.185, CI = [0.148, 0.222]
Oort, 2011: FPR = 0.170, CI = [0.140, 0.200]
Randell, 2013: FPR = 0.329, CI = [0.270, 0.388]
Rozen, 2008: FPR = 0.080, CI = [0.049, 0.111]
Rozen, 2010: FPR = 0.098, CI = [0.083, 0.113]
Terhaar sive Droste, 2012: FPR = 0.179, CI = [0.140, 0.218]
Young, 2020: FPR = 0.747, CI = [0.732, 0.762]

15µg Hb/g - Mixed

Castro, 2013: FPR = 0.058, CI = [0.038, 0.078]
Hazazi, 2010: FPR = 0.060, CI = [0.044, 0.076]
Kovarova, 2012: FPR = 0.152, CI = [0.119, 0.185]
Oort, 2011: FPR = 0.126, CI = [0.104, 0.148]
Rozen, 2008: FPR = 0.057, CI = [0.030, 0.084]
Rozen, 2010: FPR = 0.070, CI = [0.058, 0.082]

20µg Hb/g - Asymptomatic

Hernandez, 2014: FPR = 0.067, CI = [0.048, 0.086]
Liles, 2018: FPR = 0.054, CI = [0.046, 0.062]
Masau, 2021: FPR = 0.057, CI = [0.044, 0.070]
Park, 2010: FPR = 0.061, CI = [0.039, 0.083]

20µg Hb/g - Mixed

Auge, 2016: FPR = 0.145, CI = [0.094, 0.196]
Auge, 2018: FPR = 0.166, CI = [0.131, 0.201]
Castro, 2013: FPR = 0.043, CI = [0.025, 0.061]
Hazazi, 2010: FPR = 0.046, CI = [0.032, 0.060]
Kovarova, 2012: FPR = 0.130, CI = [0.096, 0.164]
Oort, 2011: FPR = 0.102, CI = [0.082, 0.122]
Randell, 2013: FPR = 0.201, CI = [0.148, 0.254]
Rozen, 2008: FPR = 0.040, CI = [0.019, 0.061]
Rozen, 2010: FPR = 0.054, CI = [0.042, 0.066]
Young, 2020: FPR = 0.569, CI = [0.551, 0.587]
